# Atomically Substitutional Engineering of Transition Metal Dichalcogenide Layers for Enhancing Tailored Properties and Superior Applications

**DOI:** 10.1007/s40820-023-01315-y

**Published:** 2024-01-23

**Authors:** Zhaosu Liu, Si Yin Tee, Guijian Guan, Ming-Yong Han

**Affiliations:** 1 Institute of Molecular Plus, Tianjin University, Tianjin, 300072 People’s Republic of China; 2 Institute of Materials Research and Engineering, A*STAR, Singapore, 138634 Singapore

**Keywords:** Transition metal dichalcogenides, Atomic substitution, Tailored structure, Tunable bandgap, Enhanced applications

## Abstract

Transition metal dichalcogenides (TMDs) are a promising class of layered materials in the post-graphene era, with extensive research attention due to their diverse alternative elements and fascinating semiconductor behavior. Binary MX_2_ layers with different metal and/or chalcogen elements have similar structural parameters but varied optoelectronic properties, providing opportunities for atomically substitutional engineering via partial alteration of metal or/and chalcogenide atoms to produce ternary or quaternary TMDs. The resulting multinary TMD layers still maintain structural integrity and homogeneity while achieving tunable (opto)electronic properties across a full range of composition with arbitrary ratios of introduced metal or chalcogen to original counterparts (0–100%). Atomic substitution in TMD layers offers new adjustable degrees of freedom for tailoring crystal phase, band alignment/structure, carrier density, and surface reactive activity, enabling novel and promising applications. This review comprehensively elaborates on atomically substitutional engineering in TMD layers, including theoretical foundations, synthetic strategies, tailored properties, and superior applications. The emerging type of ternary TMDs, Janus TMDs, is presented specifically to highlight their typical compounds, fabrication methods, and potential applications. Finally, opportunities and challenges for further development of multinary TMDs are envisioned to expedite the evolution of this pivotal field.

## Highlights

- Atomically substitutional engineering in binary transition metal dichalcogenides (TMDs) enables the facile production of ternary or quaternary TMDs with tunable (opto)electronic properties spanning the entire compositional spectrum.
- A comprehensive overview is provided on multinary TMDs, including Janus-type structures, aiming to elaborate on their theoretical foundations, synthetic strategies, tailored properties, and superior applications.
- The challenges and opportunities faced in accelerating the exploitation of multinary TMDs as highly promising nanomaterials are discussed.

## Graphical abstract

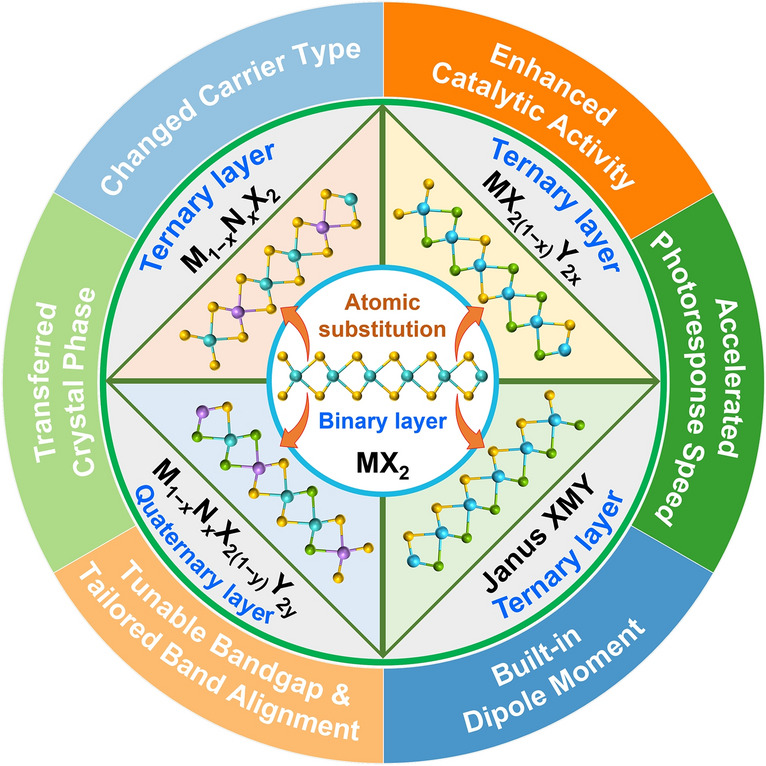

## Introduction

The discovery and in-depth investigation of graphene have led to considerable attention toward two-dimensional (2D) nanomaterials as a new family in the materials world, with potential applications in medical treatment, environmental detections, energy exploitation, and photoelectric devices [1–8]. Numerous 2D members have emerged in the post-graphene era, including transition metal dichalcogenides (TMDs), black phosphorus, hexagonal boron nitride, graphitic carbon nitride, and more [9–14]. With the comprehensive exploitation of diverse 2D nanomaterials, finding and tuning their novel features has become a goal to broaden and facilitate their utilization. However, 2D nanomaterials with fixed properties are incapable of satisfying the advanced application scenarios in efficiency, stability, specificity, and operability [15–19]. To improve their performance, elemental doping and other nanomaterials-involved hybridization have been greatly developed [20–23]. Elemental doping is a tried-and-tested strategy to modulate the band structure of 2D nanomaterials and endows tailored properties toward varied (opto)electronic devices, although the doping rate is usually restricted to < 10%, leading to a limited ability for modulating properties by introducing defects or other elements [10, 24, 25]. Alternatively, nanostructured hybridization is realized via surface adsorption of 2D nanomaterials or covalent linkage with other nanostructures to form heterojunction for more or better functionalities [19, 26, 27]. However, it is challenging to intrinsically modulate the nature of 2D nanomaterials via external contact, and the formed interface between two hybrid species exhibits a high transfer barrier for carriers, compromising the responsiveness and reaction rate of the hybridized compounds [28–31].

**Fig. 1:**
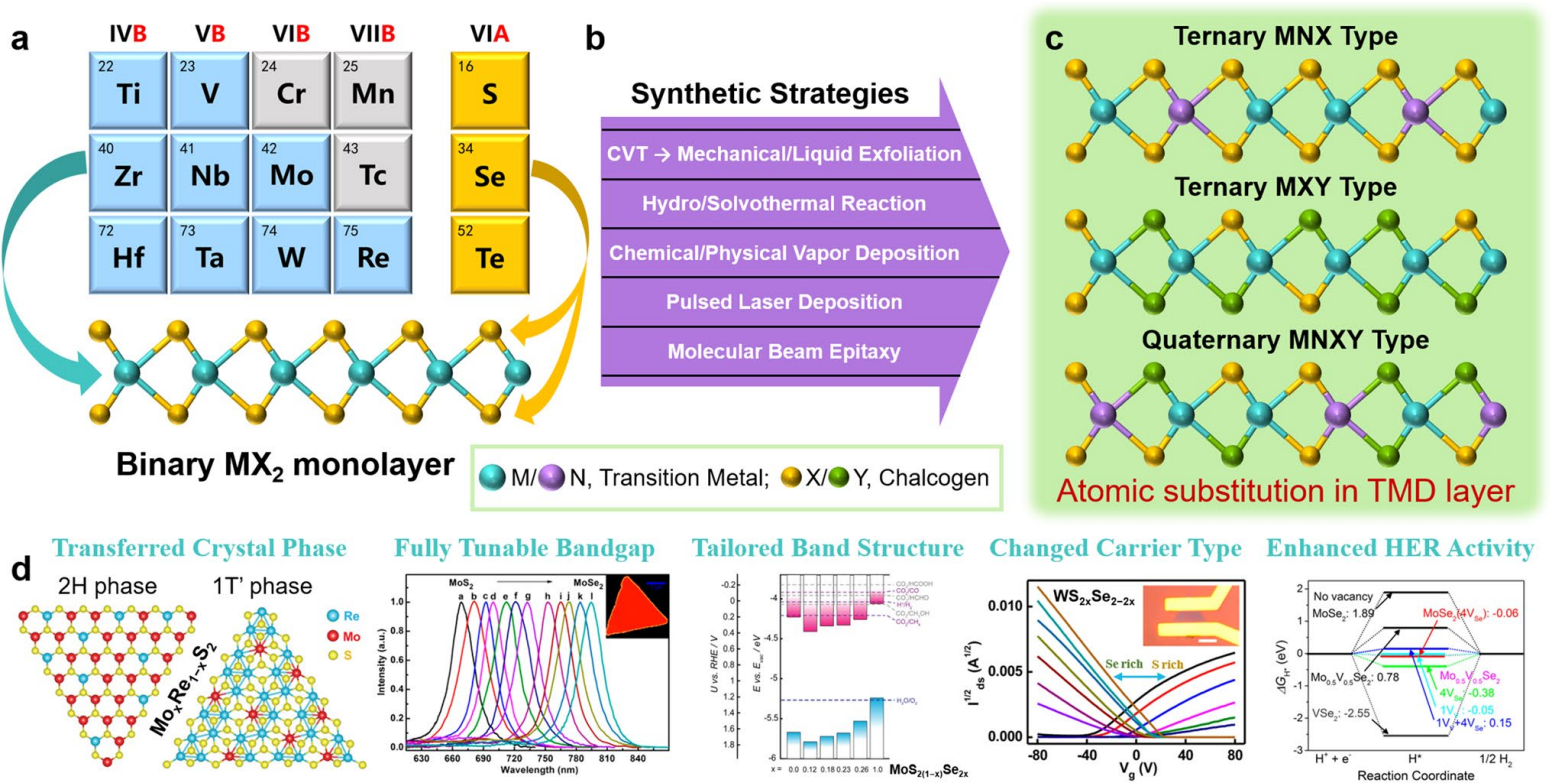
**a** Construction of binary TMD monolayer, **b** synthetic strategies for atomic substitution in TMD layer, **c** representative structures after partial modification of metal (M, N) or/and chalcogen (X, Y) atoms in TMDs, and **d** tunable/enhanced properties and improved performance provided by atomic substitution in TMD layers

TMD monolayers consist of three layers of atoms with a transition metal layer sandwiched between two chalcogen layers (i.e., X–M–X), as shown in Fig. 1a. The transition metal typically ranges from Group IVB to VIIB, while the chalcogen is S, Se, or Te in Group VIA. Many types of binary compounds with a chemical formula of MX_2_ are included in 2D TMDs, such as MoS_2_, WS_2_, MoSe_2_, and MoTe_2_. These TMD materials exhibit diverse optoelectronic properties from semiconductivity, semi-metallicity, metallicity to superconductivity, depending on their chemical composition and/or crystal phase, despite similar structural parameters [32–37]. Recently, the evolution in properties for these binary TMDs has inspired scientists to develop new degrees of freedom for tuning/optimizing the properties of 2D TMDs “from the inside out” by partly substituting the transition metal with ones from the same or adjacent group and/or the chalcogens with another type of chalcogen atom, ultimately forming ternary or quaternary TMD layers (Fig. 1b, c).

With good miscibility, atoms from the same or adjacent group can be introduced in binary TMDs at any percentage, and the resulting ternary or quaternary TMD layers maintain their structural integrity and homogeneity to easily obtain a series of fully composition-tunable structural and (opto)electronic properties between the two end-TMDs (Fig. 1d) [38–43]. For instance, the optical bandgap of MoS_2*x*_Se_2(1−*x*)_ monolayers can be continuously tuned from 1.557 to 1.856 eV as the S composition increases [44]. In monolayer Mo_*x*_Re_1−*x*_S_2_, the strong band bowing effect extends the photoresponse wavelength from visible light (633 nm) to NIR light (1,550 nm) [45]. After combining two TMDs with different carrier types in WS_2*x*_Se_2−2*x*_ monolayers, the conductive behavior is gradually regulated from *p*-type to weak bipolar and then to *n*-type with increasing S composition [46]. As the filling of d orbitals in multinary TMDs changes continuously with the composition, phase engineering of 2D TMDs can be readily achieved via atomic substitution (e.g., WSe_2(1−*x*)_Te_2*x*_ exhibits tunable crystal phases from semiconducting 2H phase to metallic 1T’ phase as *x* increases) [47, 48]. Recently, Liu’s team even observed superconductivity and Weyl semi-metallic features in Mo-rich Mo_0.8_W_0.2_Te_2_ and W-rich Mo_0.2_W_0.8_Te_2_ layers, respectively [49]. Meanwhile, ternary Janus TMD monolayers with different chalcogens on the top and bottom side of the transition metal break the crystal mirror symmetry to generate permanent built-in vertical dipole moments [50, 51].

Ternary or quaternary TMD layers have emerged as a promising upgraded material compared to pristine binary TMDs, offering significantly improved performance and exotic behaviors due to their tunable and tailored properties. For example, MoS_2_-based photodetectors show excellent photoresponsivity with a relatively long response time, while the introduction of tin to form Mo_1−*x*_Sn_*x*_S_2_ layers can modulate the defect-induced trap states to tremendously shorten the photoresponse time [52]. Atomically substitutional engineering can also enhance the catalytic activity in hydrogen evolution reaction (HER) by decreasing the adsorption-free energy of TMDs toward hydrogen, and the relatively big lattice mismatch can form vacancies on the basal plane to provide more catalytic active sites [53–55]. Recently, more and more multinary TMDs with controlled composition in special crystalline structures have been successfully fabricated to exhibit unique and excellent properties for better applications, fuel immense interest, and further research in the interdisciplinary realm of materials, physics, chemistry, and electronics. This review provides a systematic overview of recent advancements in burgeoning ternary and quaternary TMD layers. It begins with the concept of atomically substitutional engineering, classification of TMDs, substituting principles/rules, and synthetic strategies. The paper then elaborates on various tunable properties and exotic behaviors of multinary TMD layers, followed by recent exploitation on novel/enhanced applications of multinary TMDs. As an emerging ternary TMD, Janus TMDs are also presented, from their theoretical foundation to fabrication approaches and potential applications. Finally, the challenges facing TMDs are discussed in detail, and opportunities in this pivotal field are envisioned to navigate further exploration and investigation of multinary TMDs in the future.

## Concepts and Rules for Atomic Substitution in TMDs

Various functionalization strategies have been developed to construct novel 2D nanosystems for next-generation high-performance devices, including elemental doping, hybridization with other nanomaterials, and atomic substitution in TMDs (also known as alloyed TMDs and abbreviated as ATMDs). Table 1 summarizes their individual characteristics and main advantages. Elemental doping allows for a wide variety of available elements, but the proportion of introduced atoms is typically restricted to less than 10% due to lattice mismatch [10, 24, 25]. Heterojunctions are established via surface adsorption or covalent linkage in hybrids of TMDs with diversified nanostructures, allowing for new carrier separation and transfer channels that enhance the application of 2D TMDs in catalysis [19, 27]. However, the heterogeneous interface can present a high transfer barrier for carriers, compromising hybrid compound responsiveness and reaction rate. In comparison, atomic substitution in TMDs offers the ability to form random solid solutions with different transition metals from the same or adjacent group and/or different chalcogens. This form of substitution can easily regulate the properties of 2D TMDs “from the inside out” via the creation of ternary or quaternary TMDs. The resulting multinary TMDs introduce a full range of compositions in TMDs while maintaining crystal integrity and homogeneity so that crystal phase, bandgap, band alignment/structure, carrier type/density, basal-plane activity, and electroconductibility (metallicity, semiconductivity, or Wyle semi-metallicity) can be flexibly tuned [41, 45, 46, 48, 56–59]. The multinary atomic layers of TMDs not only expand the family of 2D nanomaterials, but also unlock the exotic physical and chemical properties of TMDs for greatly improved performance in optoelectronic applications.

**Table 1:** Description and comparison of functionalizations on 2D TMDs and their impacts

Functionalizations	Involved species	External ratio	Description	Enhanced properties and performances
Doping	TMDs and other elements^a^	< 10%	Trace amount of atoms in lattice or surface adsorption	Changed carrier type/density; limited tuning of bandgap and band structure; high reactive ability
Hybridization	Different types of TMDs; TMDs and other nanomaterials (0D to 2D)	20–80%	Vertical/lateral heterostructure via stacking, surface adsorption or covalent linkage	New channel for separating and transferring carriers; high catalytic activity
Atomic substitution	Ternary or quaternary TMDs	0–100%	Uniform random solid solution; structural integrity and homogeneousness	Modulated crystal phase; continuously tunable bandgap; tailored band alignment/structure; controlled carrier type/density; adjusted electroconductibility; increased surface activity

^a^The choice of element for incorporation into TMDs can be virtually any element from the Periodic Table of Elements, with the exception of elements belonging to the same or adjacent group as the elements presented in the TMDs

To achieve atomic substitution in binary TMD layers (MX_2_, Fig. 1a), an additional transition metal element and/or a different chalcogen element is introduced into a TMD layer at any proportion while maintaining the layered structure. This results in ternary or quaternary TMDs. In contrast to the case of binary TMDs with the chemical formula MX_2_, ternary TMDs can be classified as M_1−*x*_N_*x*_X_2_ (MNX type) and MX_2(1−*x*)_Y_2*x*_ (MXY type), where M/N represents different transition metal elements from Group IVB, VB, VIB, or VIIB, and X/Y represents different chalcogen elements (Fig. 1c). Additionally, there are emerging MXY-type TMDs with a highly ordered Janus structure of TMDs. These Janus TMDs contain different chalcogen elements on the top and bottom of the middle metal layer, such as S–Mo–Se [50]. This unique configuration breaks the out-of-plane structural symmetry, generating permanent built-in vertical dipole moments (see the detailed demonstration in Sect. 6) [50, 60]. It is worth noting that Sn-based metal dichalcogenides (for example, SnS_2_) have a similar layered crystal structure as TMDs, making Sn a promising candidate for atomic substitution in binary TMDs with outstanding optoelectronic performance, as reported previously [52, 57, 61–64].

**Fig. 2:**
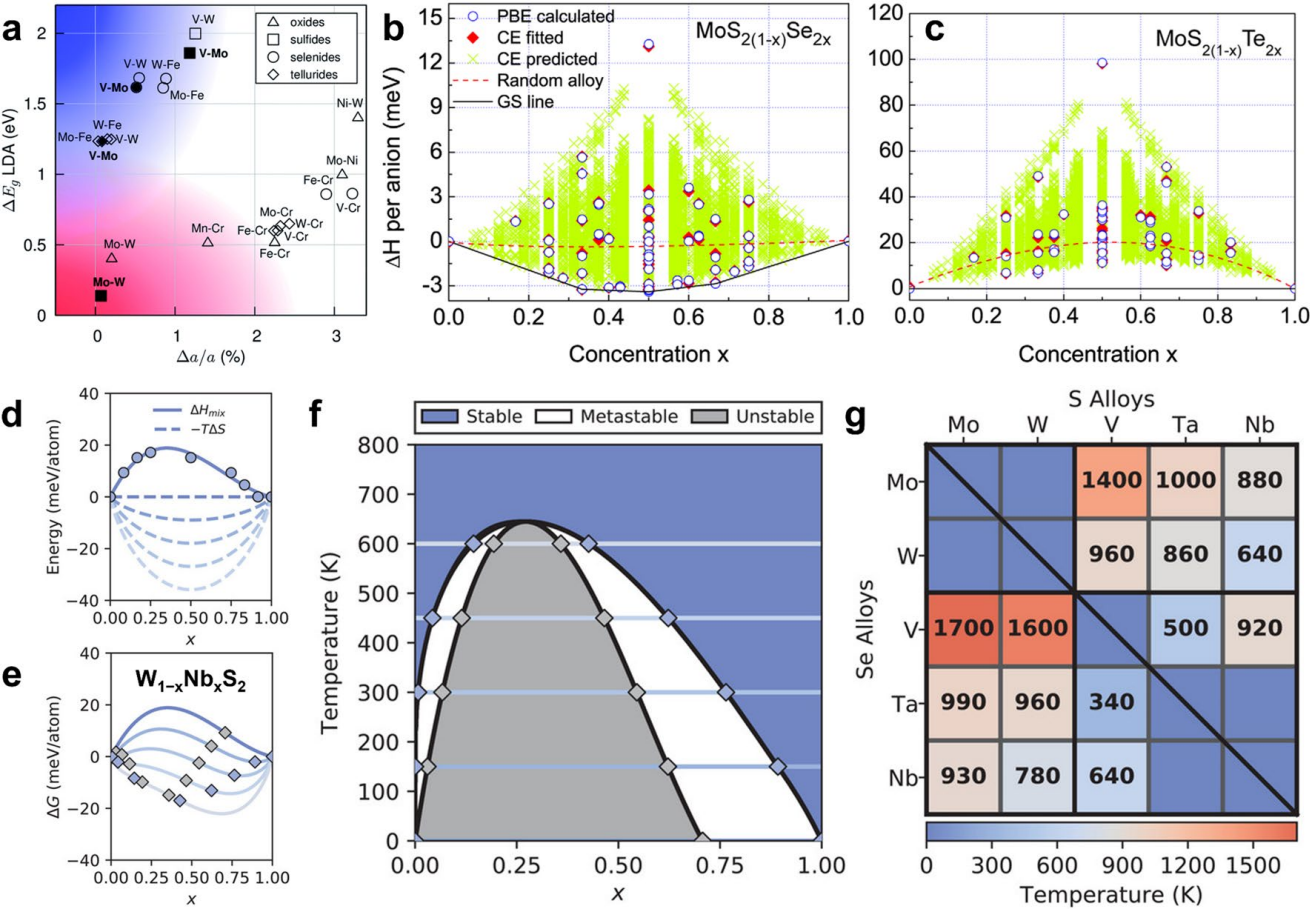
Principles/rules and theoretical guidance for synthesizing ternary TMDs. **a** Lattice constant matching for metal–metal pairs of 2H TMDs based on lattice constants. The shaded blue area in the top left corner is populated with vanadium–molybdenum and vanadium–tungsten dichalcogenides, which are metal–semiconductor alloys. The shaded pink area in the bottom left corner contains semiconductor–semiconductor alloys of molybdenum and tungsten dichalcogenides. Reproduced with permission from Ref. [65]. Copyright 2014, Royal Society of Chemistry. Calculated mixing enthalpies for **b** MoS_2(1−*x*)_Se_2*x*_ and **c** MoS_2(1−*x*)_Te_2*x*_ via different simulation methods (all symmetry-inequivalent alloy configurations in a 24-atom cell are used for the calculation by CE). Reproduced with permission from Ref. [66]. Copyright 2013, AIP Publishing. **d** DFT-calculated and fitted mixing enthalpy (Δ*H*_mix_, solid line) of W_1−*x*_Nb_*x*_S_2_, along with the entropy contribution (Δ*S*, dashed line) to the free energy at temperatures between 0 and 600 K. **e** x-dependent free energy of W_1−*x*_Nb_*x*_S_2_ obtained by merging the enthalpy and entropy terms from **d**. **f** Horizontal lines “one-to-one” correspond with the free energies in **e** for revealing stability. The blue and gray diamonds correspond to boundary points between different stability regions in the equilibrium phase diagram. **g** Miscibility temperatures of all the 20 MNX- and 5 MXY-type TMDs in the 2H phase. The top right and bottom left triangles correspond to sulfide and selenide TMDs, respectively, while the diagonal corresponds to MXY-type TMDs. Dark blue spaces indicate miscible TMDs. Reproduced with permission from Ref. [68]. Copyright 2020, Wiley–VCH

Before designing and fabricating multinary TMD layers, it is crucial to assess the possibility of atomic substitution theoretically with considering a wide range of atomic incorporation from 0 to 100% in experiments. In evaluating the feasibility, parameters such as lattice constant and bandgap of the two types of TMDs for the final ternary or quaternary atomic layers, also known as end-TMDs, must be taken into account. For instance, MoS_2_ (*x* = 0) and WS_2_ (*x* = 1) serve as the end-materials of Mo_1−*x*_W_*x*_S_2_ layers. Yakobson’s group provided a set of rules to choose possible candidates for end-materials, which includes: (1) |*a*_1_ − *a*_2_|/max{*a*_1_, *a*_2_} < 0.034, (2) Δ*d*_*M*–*X*_ < 0.1 Å, (3) (*E*_g1_ > 0) or (*E*_g2_ > 0) [65]. Here, a_1_ and a_2_ represent the lattice constants of the two end-materials, Δ*d*_*M*–*X*_ is the difference in bond length between metal−chalcogen, E_g1_ and E_g2_ are the bandgaps of the two end-TMDs, respectively. The first and second rules require a good match in lattice constants and metal–chalcogen bond distances between the two end-materials. Ternary TMDs that consist of two end-materials with large lattice mismatches and even different crystal structures often exhibit poor miscibility and a tendency to separate or segregate into distinct phases. This phase separation has detrimental consequences for their intrinsic properties and their applications in devices. To address this issue, it is necessary to elevate the temperature sufficiently to improve the miscibility or solubility of the ternary TMDs [66, 67]. Meanwhile, the third rule guarantees that at least one of the end-materials is semiconducting for tunable bandgap achievement. Based on these rules, the pairs of end-materials are summarized in Fig. 2a according to the differences in theoretical bandgap and lattice constant [65]. The data points on the left side correspond to relatively small lattice mismatch, making them promising candidates for multinary TMDs. Additionally, the data points located in the top left (blue shade) signify that the two end-TMDs are metallic and semiconducting, respectively, leading to a fascinating metal–semiconductor phase transition as *x* changes.

To assess the thermodynamic stability of miscible ternary TMD layers, the mixing enthalpy (Δ*H*_mix_) can be used as an important reference for variable lattice matching degrees (Δ*d*_*M*–*X*_) [66, 67]. Over the past few years, the cluster expansion (CE) method has been used to calculate the mixing enthalpies of diverse ternary TMDs, allowing for an assessment of their thermodynamic stability through the full microscopic description of atomic configurations in a crystal [42, 65, 66]. Studies from first-principles simulation together with the CE method have calculated the Δ*H*_mix_ of 2H-phase Mo- and W-based MXY-type TMD monolayers with different compositions and configurations [66]. As seen in Fig. 2b, the Δ*H*_mix_ of the MoS_2(1−*x*)_Se_2*x*_ random alloys at all compositions are almost negative (red dashed line), indicating that the ternary TMDs are stable and that random solid solutions are favored at 0 K. This phenomenon can be attributed to the small lattice mismatch between the two end-materials and the energy gain induced by the large additional charge exchange in such alloys [66, 67, 69, 70]. Similar calculations showed that the Δ*H*_mix_ of WS_2(1−*x*)_Se_2*x*_ and Mo_1−*x*_W_*x*_S_2_ random alloys at all compositions were also confirmed to be negative [65, 66]. This makes it easy to synthesize and preserve such ternary TMDs with complete miscibility in some practical experiments [42, 59, 66, 68, 71–73]. On the other hand, the Δ*H*_mix_ of MoSe_2(1−*x*)_Te_2*x*_, WSe_2(1−*x*)_Te_2*x*_, MoS_2(1−*x*)_Te_2*x*_ (Fig. 2c), and WS_2(1−*x*)_Te_2*x*_ random alloys are all positive due to relatively large lattice mismatch and different crystal phases between the two end-materials [66]. Thus, these ternary TMDs have no energetically favorable configuration/structure at 0 K and will undergo phase separation/segregation, requiring higher temperatures to improve their miscibility/solubility [48, 67, 74, 75].

It has been found that single-phase random solid solutions can also be obtained for ternary TMDs with positive Δ*H*_mix_ at sufficiently high temperatures due to the large entropic energy gain [66, 69]. Indeed, the stability of ternary TMDs depends on the change in Gibbs free energy of mixing (Δ*G*_mix_), which can be expressed by Δ*G*_mix_(*x*; *T*) = Δ*H*_mix_(*x*) − *T*Δ*S*(*x*) [68]. When Δ*H*_mix_ is greater than zero, Δ*G*_mix_ can be reduced to a negative value by increasing temperature because the entropy (Δ*S*) is positive [66, 68, 75]. Therefore, the calculation and evaluation of enthalpy and entropy are crucial prerequisites for obtaining stable and single-phase uniform ATMDs. For example, Amin and his colleagues used density functional theory (DFT) to calculate the Δ*H*_mix_ and the temperature-dependent − *T*Δ*S* of the random 2H-phase W_1−*x*_Nb_*x*_S_2_ monolayer (Fig. 2d–e) [68]. Their results showed that as the temperature increases, the entropy term gradually dominates to reduce the Δ*G*_mix_, making the W_1−*x*_Nb_*x*_S_2_ random alloys more stable. Based on the analysis of temperature-dependent stability (equilibrium phase diagram), the composition of an ATMD at a given finite temperature can be divided into three regions: stable, metastable, and unstable (Fig. 2f). At 640 K, the three regions meet, indicating that single-phase W_1−*x*_Nb_*x*_S_2_ random solid solutions are stabilized at arbitrary composition (i.e., the miscibility temperature). As summarized in Fig. 2g, the miscibility temperatures of 20 MNX-type and 5 MXY-type 2H-phase ATMDs were systematically calculated using first-principles DFT modeling and were further confirmed by experimental synthesis. These findings and discussions indicate that the theoretical work provides valuable guidance for producing random and uniform/homogeneous ATMDs.

In the fabrication of 2D ATMDs, high temperature is generally required and atomic substitution in TMDs can induce lattice distortion to increase their configurational entropies. This often results in the production of ATMDs in a random phase rather than a separated phase [76]. Meanwhile, the degree of alloying/uniformity of the synthesized ATMDs varies depending on the used precursors, synthetic strategies, and experimental conditions [45, 55, 65, 68, 77, 78]. In addition to thermodynamic stability, atomic distribution and electronic properties are also significant factors that influence the crystal phase of 2D ATMDs due to their different filling of the d orbitals [39, 45, 47, 77]. Dynamic stability should also be considered during the fabrication and utilization of 2D ATMDs. Through molecular dynamics (MD) simulations, Ajayan and his colleagues investigated the effect of composition (*x*) of Mo_*x*_W_1−*x*_S_2_ alloys on their mechanical stability and found that the alloying of MoS_2_ can withstand higher stress values but reduces its failure strain [79].

## Strategies and Approaches for Atomic Substitution in TMD Layers

Once the feasibility of multinary TMDs has been ensured using the aforementioned rules, atomic substitution in binary TMD layers can be achieved through two approaches: top–down and bottom–up. In the top–down approach, massive layered ATMD bulky crystals are first synthesized and then exfoliated into atomically thin layers [48, 80, 81]. This approach includes mechanical exfoliation and liquid exfoliation. In contrast, the bottom–up approach involves synthesizing single-/few-layer ATMD nanosheets directly from appropriate precursors. This approach includes hydro/solvothermal reaction, chemical vapor deposition, pulsed laser deposition, and so on [52, 82–86]. The synthesis of parent bulky crystals in the top–down approach typically requires high temperatures and a lengthy chemical vapor transport (CVT) process (~ 1 week). Although the resulting bulk ATMDs have high crystal quality, to obtain atomically thin (monolayer or few-layer) ATMDs requires an additional manual micromechanical or liquid exfoliation process, which introduces randomness in the layer number, morphology, and size of exfoliated ATMD layers, limiting their scaling-up applications [3, 78, 87–89]. To achieve highly controllable synthesis of various 2D ATMDs, bottom–up approaches can utilize diversified chemical precursors and precisely adjustable experimental conditions to fabricate numerous 2D ATMDs with high quality and excellent performance. In this discussion, we will mainly focus on the bottom–up approaches after briefly describing liquid exfoliation.

### Liquid Exfoliation

**Fig. 3:**
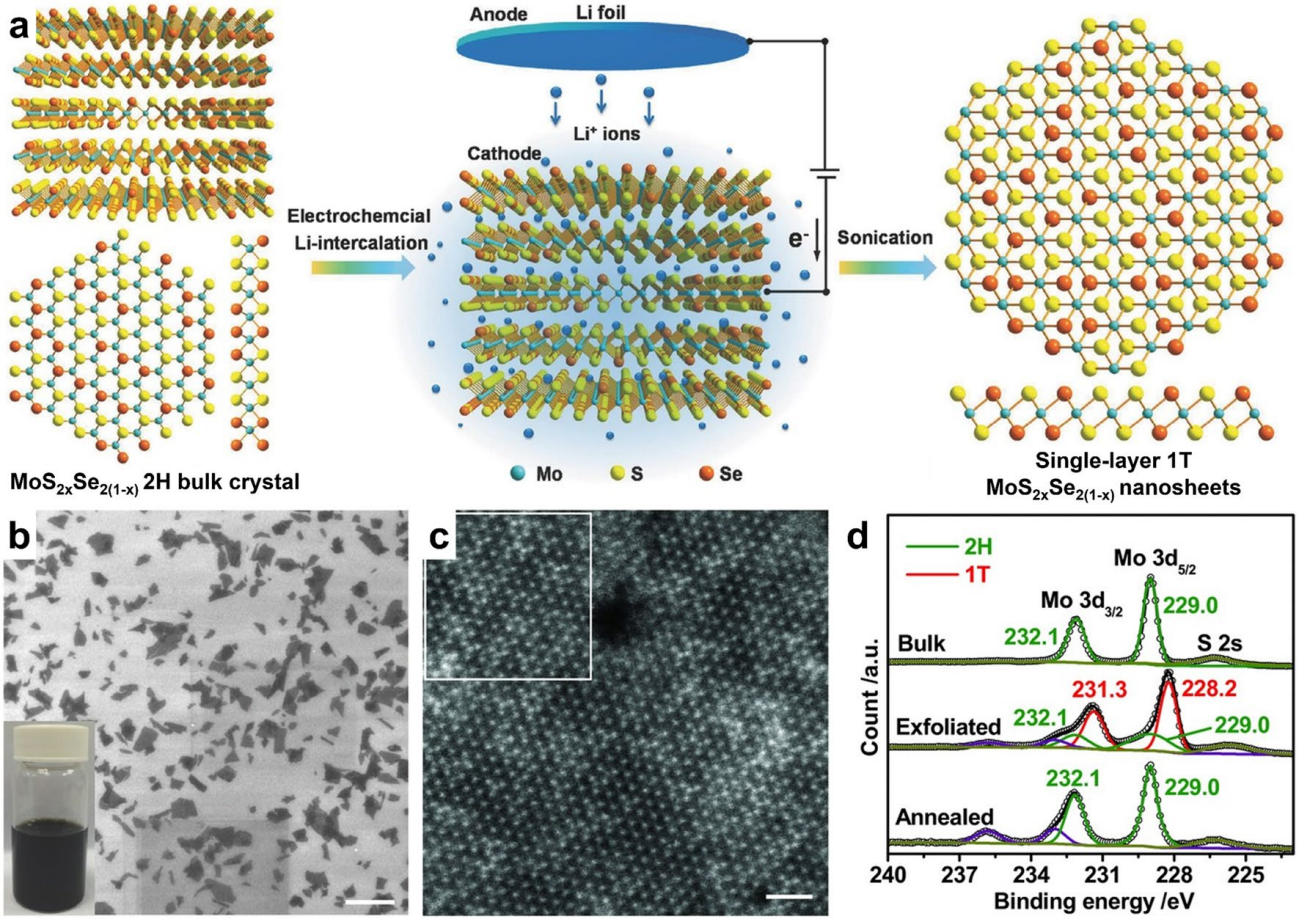
Li-ion intercalation-assisted liquid exfoliation of 2H-phase MoS_2*x*_Se_2(1−*x*)_ bulk crystal to form 1T-phase MoS_2*x*_Se_2(1−*x*)_ monolayer. **a** Schematic illustration for preparing single-layered MoS_2*x*_Se_2(1−*x*)_ nanosheets. **b–d** Characterization of MoS_2*x*_Se_2(1−*x*)_ nanosheets for demonstrating the formation of 1T-phase in the exfoliated MoS_2*x*_Se_2(1−*x*)_ nanosheets: **b** SEM image of exfoliated high-concentration MoS_2*x*_Se_2(1−*x*)_ nanosheets (scale bar, 2 µm). **c** Atomic STEM image of a typical MoS_2*x*_Se_2(1−*x*)_ nanosheet with 1T-phase (scale bar, 1 nm). **d** High-resolution XPS Mo 3d spectrum of 2H-phase MoS_2*x*_Se_2(1−*x*)_ bulk crystal, and the exfoliated (1T) and annealed (2H) MoS_2*x*_Se_2(1−*x*)_ nanosheets. Reproduced with permission from Ref. [80]. Copyright 2016, Wiley–VCH

Both sonication-assisted and ion intercalation-assisted methods of liquid exfoliation are capable of producing dispersive nanosheets in liquid with high yield, while also meeting the requirements of low cost and simple operation [90, 91]. During sonication-assisted liquid exfoliation, ultrasonic waves induce bubbles between layers of bulk crystals, and the micromechanical forces generated by the bursting of these bubbles drive the materials to be exfoliated into monolayers [3, 92]. By contrast, the ion intercalation-assisted liquid exfoliation method involves the insertion of small metal ions (such as Li^+^ or Na^+^) into the layered crystals to expand the interlayer spacing, which weakens the van der Waals interaction between adjacent layers and facilitates the separation of ATMD layers [3, 53, 90]. Interestingly, the introduction of small metal ions into ATMD layers can increase the electron density of the d orbitals of transition metals, leading to a phase transformation from 2H to 1T [39, 53]. As illustrated in Fig. 3, a Li-ion intercalation-assisted liquid exfoliation method was developed using LiPF_6_ as the electrolyte, which allowed for the preparation of a high concentration (~ 66%) of metallic 1T-phase MoS_2*x*_Se_2(1−*x*)_ and Mo_x_W_1−*x*_S_2_ nanosheets from their 2H-phase bulk crystals [80].

### Hydrothermal and Solvothermal Reaction

**Fig. 4:**
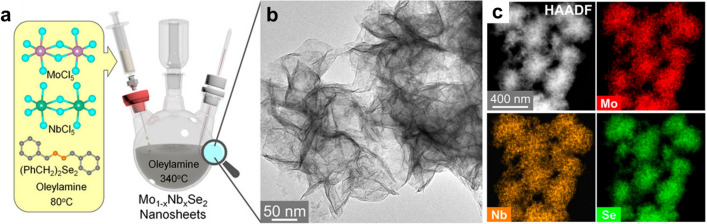
**a** Schematic diagram for the synthesis of Mo_1−*x*_Nb_*x*_Se_2_ nanosheets through one-pot solvothermal reaction using MoCl_5_, NbCl_5_, (PhCH_2_)_2_Se_2_ as precursors with different ratios and oleylamine as solvent. **b** HRTEM image for showing the assembly of nanosheets into flower-like spheres. **c** HAADF-STEM image and EDX elemental mapping of Mo (L shell), Nb (L shell), and Se (L shell) for Mo_0.5_Nb_0.5_Se_2_. Reprinted with permission from Ref. [93]. Copyright 2021, American Chemical Society

Hydro/solvothermal reactions involve mixing preselected precursors (such as metal salts and chalcogens) and appropriate solvents in a closed reactor, under specific experimental conditions. By manipulating the relative amounts of reactants and controlling various experimental factors such as temperature, precursor state, and surfactant, ATMDs can be fabricated with adjustable compositions (ranging from 0 to 1), tunable sizes, and multifarious morphologies. For instance, Kang’s team utilized chloride (MoCl_5_ and NbCl_5_) or sodium salts (Na_2_MoO_4_ and NaReO_4_) as metal precursors to synthesize a series of ATMD nanosheets with fully tunable compositions via hydro/solvothermal reactions, including Re_1−*x*_Mo_*x*_Se_2_, Re_1−*x*_Mo_*x*_S_2,_ and Mo_1−*x*_Nb_*x*_Se_2_ (as illustrated in Fig. 4) [55, 93, 94]. The reactions were conducted at relatively low temperatures of ~ 300 °C, which resulted in such ATMDs being prone to phase segregation and domain separation, and annealing treatments were required to ameliorate their miscibility. Interestingly, both experimental results and theoretical simulations demonstrated that a certain degree of phase separation can promote the formation of atomic vacancies, significantly enhancing their electrocatalytic activity [93].

### Chemical Vapor Deposition

**Fig. 5:**
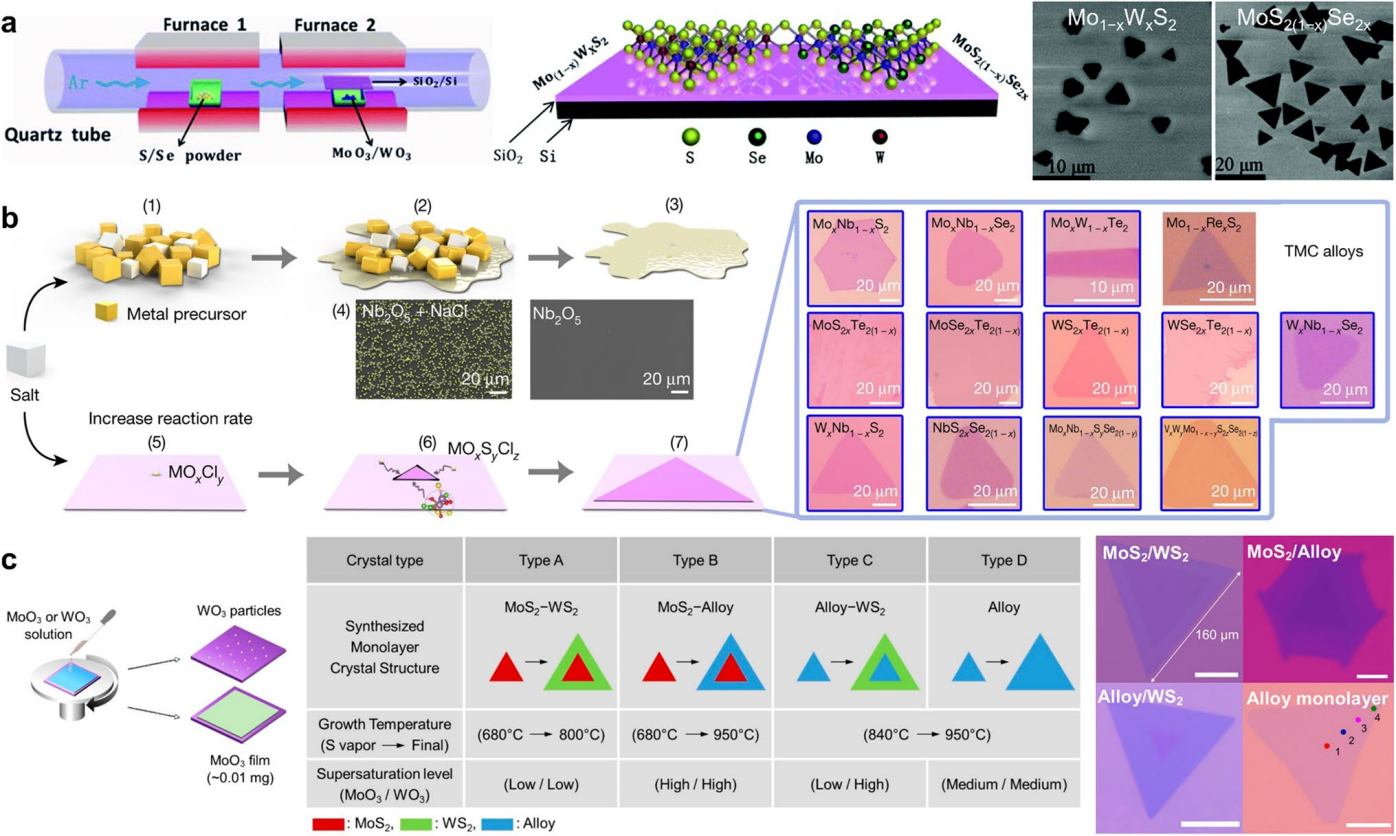
Schematic diagrams of a typical CVD process for the controllable fabrication of high-quality ATMD monolayers. **a** Typical tube-furnace set-up employed for the growth of ternary monolayers (left figure), the two-dimensional structures of Mo_(1−*x*)_W_*x*_S_2_ monolayer and MoS_2(1−*x*)_Se_2*x*_ monolayer (middle figure), and the corresponding SEM morphology of Mo_(1−*x*)_W_*x*_S_2_ and MoS_2(1−*x*)_Se_2*x*_ (right figure). Reprinted with permission from Ref. [95]. Copyright 2015, Royal Society of Chemistry. **b** Proposed process for decreasing the melting point of the precursors after the addition of salt, the growth process of the 2D atomic layer, and the optical images of 13 ATMD layers synthesized using the molten-salt-assisted CVD method. The SEM images of the Nb nucleus with and without salt are also presented. Right figures are optical images of 13 ATMD layers synthesized using the molten-salt-assisted CVD method. Reprinted with permission from Ref. [33]. Copyright 2018, Nature Publishing Group. **c** Solution-processed precursor deposition for the CVD growth of MoS_2_/WS_2_ lateral heterostructures and alloys (left), the reaction conditions for the CVD growth of each monolayer structure (middle), and the optical images of each monolayer structure (right). Reprinted with permission from Ref. [106]. Copyright 2019, American Chemical Society

The chemical vapor deposition (CVD) process is commonly executed in a tubular furnace divided into multiple temperature zones. Diverse precursors and substrates, such as SiO_2_/Si, mica or Al_2_O_3_, are positioned in different temperature zones. The high-temperature vaporized precursors are transported to a specific location using a carrier gas to initiate the growth of 2D ATMDs via reaction/deposition. The excess carrier gas and precursors are subsequently discharged out of the tube, as illustrated in Fig. 5a [95–97]. By adjusting key growth parameters like precursors (type, state, and pretreatment mode), promoters/additives, substrates, and gas flow, precisely controlled growth of various ATMD layers can be achieved with fully tunable compositions, high quality, and adjustable size/morphology/layer number [4, 96, 98–100]. The high-temperature deposition and 2D growth of CVD-fabricated ATMDs (> 700 °C) result in excellent crystallinity and uniformity/homogeneity, and the directly grown ATMDs on SiO_2_/Si substrates ensure good contact, facilitating the fabrication of high-performance (opto)electronic devices. Additionally, modified CVD methods such as additive-assisted CVD, confined-space CVD, and inductively coupled plasma-CVD have been recently developed to fabricate ATMD layers with well-crystalline quality and tailored properties [4, 33, 52, 101, 102]. In a more inspiring development, a novel technique called modularized local-precursor-supply CVD has successfully enabled the batch manufacturing of wafer-scale homogeneous multinary TMDs by supplying uniform precursors to wafers face-to-face in designed modules, providing a powerful path for the industrialized application of ATMDs, especially in (opto)electronics [103–105].

Liu’s team developed a molten-salt-assisted CVD method that has been shown to lower the melting point of metal precursors, promote the formation of intermediates, and improve the quality of ATMD monolayers by modulating the reaction rate [33]. This method was used to synthesize various high-quality ATMD monolayers, which proved to be a powerful strategy for investigating the fundamental properties of ATMDs and fabricating high-performance devices (Fig. 5b). Lee and co-workers selectively synthesized ATMD monolayers and their lateral heterostructures by combining the CVD method with a solution-processed precursor deposition [106]. By precisely controlling the timing and supersaturation degree of liquid-phase precursors, many types of lateral heterostructures were reliably grown (Fig. 5c). This work has opened up new avenues for the manufacture of advanced heterojunction devices, thanks to the tunable structural and electronic properties of ATMD layers. Xiao’s work demonstrated the synthesis of multifarious high-quality ATMD monolayers with large lateral size via a confined-space CVD method [52, 107–109]. By introducing an assistant substrate, contaminants and by-products were blocked, thereby significantly increasing the size and crystal uniformity of ATMD monolayers. To accelerate the industrialized application (such as in integrated circuits) of ATMDs, Xue et al. developed a CVD-based modularized strategy for the wafer-scale batch production of multinary TMDs, including the Janus type [103]. This strategy enables vertical stacking of multiple modules and localizes precursor within each module, allowing for the batch fabrication of multinary TMDs on wafer sizes up to 12 inches.

### Physical Vapor Deposition and Other Strategies

Physical vapor deposition (PVD) is a direct method for synthesizing ATMDs from TMDs that can be easily vaporized [46, 84, 110]. For instance, MoS_2*x*_Se_2(1−*x*)_ (*x* = 0–0.40) monolayers were synthesized successfully by using MoS_2_ and MoSe_2_ powders as precursors. The precise tuning of the alloy composition (*x*) was achieved by controlling the temperature of MoSe_2_ powders [84]. In another study, the same group introduced Se vapor during the PVD process to prevent the decomposition of MoSe_2_ to Mo_3_Se_4_ at high temperatures. This approach resulted in the successful fabrication of selenium-rich MoS_2(1−*x*)_Se_2*x*_ alloys with x ranging from 0.41 to 1.00 [110]. The lateral size and morphology of MoS_2(1−*x*)_Se_2*x*_ domains can be controlled effectively by tuning the temperature and its gradient in the deposition zone.

In addition to the typical strategies described above, several emerging and advanced approaches have demonstrated great potential for fabricating high-quality ATMDs. One such approach to achieve atomic substitution is through low-energy ion implantation or beam-mediated ion irradiation of pre-existing binary TMDs, such as CVD-grown MX_2_, allowing for highly controllable fabrication of ATMDs [111–115]. For example, Bartels and co-workers utilized an Ar^+^-ion beam to remove sulfur atoms from MoS_2_ monolayers grown on SiO_2_/Si substrates via CVD and then controlled the insertion cycles of Se precursor to produce MoS_2(1−*x*)_Se_2*x*_, achieving precise bandgap tuning [113]. Other powerful techniques such as pulsed laser deposition (PLD) and molecular beam epitaxy (MBE) have also been extended to produce high-quality multinary TMDs. For example, Yao and co-workers pioneered the fabrication of centimeter-scale and high-quality Mo_0.5_W_0.5_S_2_ alloy films using PLD for the first time [116]. This approach utilized highly pure Mo, W, and S elements as precursors and resulted in low defect density and unique alloying effects. The Mo_0.5_W_0.5_S_2_-based photodetector exhibited outstanding photoresponse performance. Song et al. reported a novel strategy for synthesizing Mo_1−*x*_W_*x*_S_2_ alloys by sulfurizing super-cycle atomic layer deposition (ALD) of Mo_1−*x*_W_*x*_O_*y*_ films [117]. The resulting Mo_1−*x*_W_*x*_S_2_ alloys exhibited high crystal quality and composition/layer number-dependent bandgaps. Furthermore, a vertically composition-controlled (VCC) Mo_1−*x*_W_*x*_S_2_ multilayer was fabricated using multiple continuous super-cycles. The resulting Mo_1−*x*_W_*x*_S_2_ multilayer-based photodetector demonstrated much higher photocurrent than MoS_2_- and WS_2_-based photodetectors. More recently, Zhang et al. synthesized V_*x*_Mo_1−*x*_Se_2_ monolayers using a MBE method and systematically investigated the evolution of thermal stability, electronic structure, and magnetism of monolayer V_*x*_Mo_1−*x*_Se_2_ as a function of V concentration (x) [86]. The results showed that *x* = 0.44 was a critical value: The alloys at *x* < 0.44 underwent semiconductor–metal phase separation, but the alloys at *x* > 0.44 preferred to form stable and homogeneous metallic phases. For better understanding of the different bottom–up methods, their strength and weakness are summarized in Table 2.

**Table 2:** The strength and weakness of different bottom–up strategies

Parameters	Hydro/solvothermal reaction	CVD	PLD	MBE
Uniformity	Medium	High	High	High
Controllability	High	High	High	High
Scalability	Medium	High	High	High
Efficiency	Medium	High	Medium	Medium
Cost effectiveness	High	Medium	Medium	Low

## Diverse Characteristics of Multinary TMDs

In contrast to binary TMDs, multinary TMD layers are created through atomically substitutional engineering that introduces a large number of heteroatoms. This results in substantially modulated properties, such as phase evolution from 2H to 1T/1T’, fully tunable bandgaps, and unique Raman shifts [44, 49, 55]. In this section, we provide an overview of the typical characteristics of multinary TMD layers, their composition-dependent features, and their great potential for various applications.

### Varied Crystal Structures of Multinary TMDs

According to reports, phase transitions in binary TMDs can be achieved through various methods such as high pressure, thermal annealing, and laser/electron-beam irradiation [39, 118]. Similarly, atomic substitution has been found to be a more mild and efficient strategy to induce phase transition/evolution from 2H to 1T/1T’ in TMD layers for enhancing the performance of 2D TMDs. Under harsh conditions, the lattice structures of binary TMDs undergo physical alterations and lattice reconstruction, leading to phase transitions. However, the resulting 1T/1T’-phase TMDs are usually metastable and tend to revert back to the thermodynamically stable 2H phase [39, 118]. In contrast, atomic substitution in TMDs allows for continuous changes in the electron filling within the d orbitals of ATMDs as the composition varies. This leads to the formation of stabilized 1T/1T’-phase ATMDs with homogeneous and integrated crystal structures, providing exciting opportunities for investigating exotic physical phenomena and developing futuristic devices [47, 119–121].

As is well-known, the two most common crystal phases in binary TMDs are the 2H phase with trigonal prismatic coordination (*D*_3h_ symmetry) and the 1T-phase with octahedral coordination (*D*_3d_ symmetry) [32, 82]. Due to the different filling in the d orbitals of transition metals, TMD nanomaterials exhibit different thermodynamically stable crystal phases and diversified conductive properties, such as semiconductivity, metallicity, and semi-metallicity [39, 119]. Group IVB (*d*^0^) and Group VIB (*d*^2^) TMDs show stable octahedral structure (1T phase) and trigonal prismatic structure (2H phase), respectively. Group VB (d^1^) TMDs have both 1T and 2H phases, while Group VIIB (*d*^3^) TMDs exhibit a distorted octahedral structure due to the distortion or/and dimerization of metal atoms (i.e., 1T’ phase) [39, 119]. Therefore, ATMD layers composed of two end-materials with different crystal phases will undergo phase transition during the continuous change of electron filling of d orbitals [47, 65]. For instance, Mo_1−*x*_Re_*x*_Se_2_ alloys will undergo a phase transition from 2H to 1T’ as *x* increases, with a turning point at *x* = 0.42 [47]. The phase structure of WTe_2*x*_S_2(1−*x*)_ alloys will transform from 2H to 1T’ at *x* = 0.45, accompanied by a transition of conductivity from semiconducting to metallic. Interestingly, Group VIB (*d*^2^) ATMDs with thermodynamically stable 2H phase still retain the intriguing metastable metallic 1T-phase [80, 122]. In recent research, alloying of TMDs was found to reduce the energy barriers for the phase transition from 2H to 1T, thus facilitating the formation of 1T-phase in Mo_1−*x*_W_*x*_S_2_ monolayer via a rapid cooling CVD process [123]. Similarly, Yang et al. synthesized Mo_1−*x*_W_*x*_S_2_ nanosheets with 1T/2H phase heterostructures via a one-step liquid-phase reaction, and the 1T/2H phase ratio can be adjusted by tuning the reaction temperature [122].

**Fig. 6:**
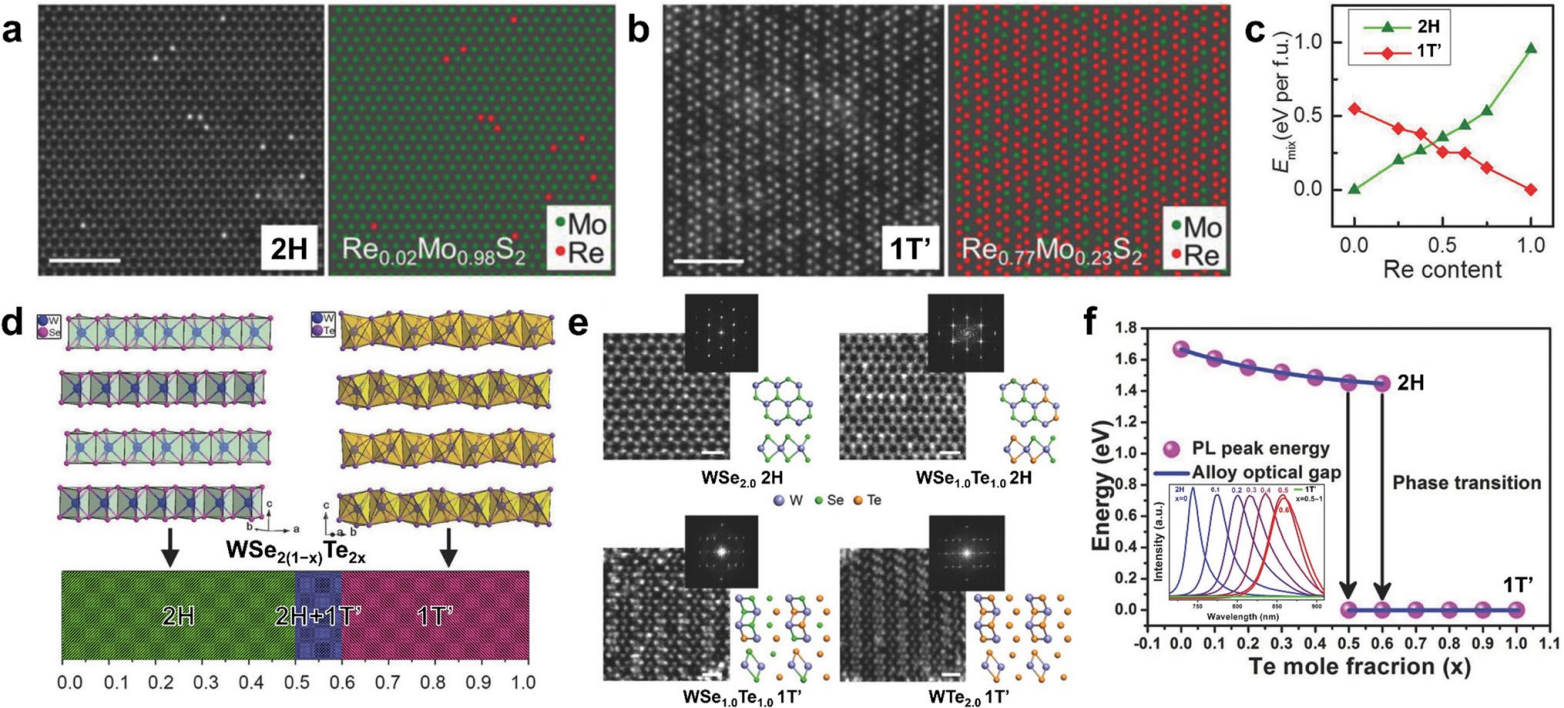
Transition of crystal phase in 2D ATMDs. **a–b** Structure of Re_*x*_Mo_1−*x*_S_2_ monolayers at different Re concentrations, as shown in the experimental STEM-ADF images (left) paired with the corresponding atom mapping images (right). To enhance clarity, S atoms are excluded from the atom mapping images. **c** Calculated mixing/formation energy in 2H- and 1T’-phase Re_*x*_Mo_1−*x*_S_2_ alloy at different Re concentrations. Reproduced with permission from Ref. [120]. Copyright 2018, Wiley–VCH. **d** Crystal structure of 2H WSe_2_ and 1T’ WTe_2_, along with the composition-dependent phases in WSe_2(1−*x*)_Te_2*x*_. **e** Atomic resolution STEM characterization of WSe_2(1−*x*)_Te_2*x*_ (*x* = 0–1) monolayers with different Te concentration. Z-contrast STEM images reveal the atomic structure of pristine WSe_2_ monolayer in the 2H phase, alloyed WSe_1.0_Te_1.0_ monolayer in the 2H phase, alloyed WSe_1.0_Te_1.0_ monolayer in the 1T’ phase, and monolayer WTe_2_ in the 1T’ phase. Corresponding FFT patterns are shown in the inset. **f** Composition-dependent band gaps (x) and the photoluminescence spectra (PL, inset) of the monolayer WSe_2(1−*x*)_Te_2*x*_ alloys. Reproduced with permission from Ref. [48]. Copyright 2016, Wiley–VCH

In addition to thermodynamics, lithium-ion intercalation has also been shown to effectively obtain 1T-phase ATMD monolayers [53, 80]. During lithiation treatment, the transition metal d orbitals’ electron density increases to destabilize the original 2H phase and increase the 1T phase’s stability, leading to a phase transition from 2H to 1T [119]. Zhang’s group, for example, prepared single-layer Mo_*x*_W_1−*x*_S_2_ and MoS_2*x*_Se_2(1−*x*)_ alloys with a high concentration of metallic 1T-phase from their 2H-phase parent bulk crystals through lithium-ion intercalation [80]. Instead of external approaches, such as those mentioned above, alloying two end-TMDs with different crystal phases can intrinsically change their thermodynamically stable crystal phase by continuously tuning the d-orbital filling, providing composition-dependent tunable crystal phases and extra fascinating properties [47]. In M_1−*x*_Re_*x*_X_2_ (*M* = Mo, W; *X* = S, Se) systems, the insertion and substitution of Re atoms introduce additional electrons into the lattice to fill the higher energy levels of MX_2_, which destabilizes the triangular prismatic 2H phase. At the same time, it increases the stability of the octahedral 1T/1T’ phase, inducing a phase transformation from 2H to 1T/1T’ [45, 47, 120, 124]. In Yang’s work, the 2H-to-1T’ phase transition was successfully achieved in Re_*x*_Mo_1−*x*_S_2_ monolayers as *x* increased, and the experimental results and DFT calculations confirmed that the phase transition occurred at approximately *x* = 0.5 (Fig. 6a–c) [120]. Similarly, Liu’s team achieved phase transitions from 2H to 1T’ in WSe_2(1−*x*)_Te_2*x*_ monolayers at *x* = 0.5–0.6 (Fig. 6d–f) [48]. This semiconductor-to-metal transition in 2D ATMDs provides an inspiring option for designing innovative functional devices with accessional fantastic properties and novel physical effects [39, 125].

Recently, the M_1−*x*_Re_*x*_X_2_ systems have demonstrated a reverse transition from 1T’ to 2H due to the significant differences in structural and electronic properties between the two end-materials. For example, Re_1−*x*_Mo_*x*_Se_2_ alloy nanosheets exhibit a phase transition from 1T’ to 2H with increasing x, and theoretical calculations show that alloying promotes the formation of atomic vacancies on the surface of alloy nanosheets, further improving HER performance [55]. Interestingly, since ReS_2_ and MoS_2_ are two semiconductors with completely different phase structures, Mo_*x*_Re_1−*x*_S_2_ alloys exhibit a much stronger bandgap bowing effect compared to Mo_1−*x*_W_*x*_S_2_ and MoS_2(1−*x*)_Se_2*x*_, significantly expanding the tunable bandgap and spectral response range in such alloys [45]. Overall, the diverse 2D TMDs with varying structural and electronic properties provide numerous choices for designing diversified 2D ATMDs with promising applications in various advanced fields. Atomically substitutional engineering in TMDs will induce lattice strain and even phase transition/evolution of ATMD layers, benefiting from their different structural properties like lattice constant and crystal phase. Table 3 summarizes the atomic substitution-induced phase transitions in specific ternary TMDs, providing inspiration for designing novel ATMD layers. In particular, the 2H-to-1T/1T’ phase transition can increase the metallicity of 2D ATMDs and introduce more active sites on their basal plane, making them irreplaceable in the realms of catalysis and gas-sensing [47, 53, 80, 120, 127]. Moreover, the tunable crystal phase feature enables ATMDs to perform better in HER and provides an inspiring opportunity for investigating new functional devices [54, 55, 120].

**Table 3:** Atomic substitution-induced phase transition in special ternary TMDs

Ternary TMDs	Phase transition as *x* increases	Turning point of phase transition	Refs
Mo_1−*x*_Re_*x*_Se_2_	2H→1T’	~ 0.42	[47]
WSe_2(1−*x*)_Te_2*x*_	2H→1T’	~ 0.5–0.6	[48]
Mo_1−*x*_V_*x*_Se_2_	2H→1T	~ 0.7	[54]
WTe_2*x*_S_2(1−*x*)_	2H→1T’	~ 0.45	[74]
Re_*x*_Mo_1−*x*_S_2_	2H→1T’	~ 0.5	[120]
W_1−*x*_V_*x*_Se_2_	2H→1T	~ 0.7	[121]
W_1−*x*_Re_*x*_S_2_	2H→1T’	~ 0.4375	[124]
Nb_1−*x*_V_*x*_Se_2_	2H→1T	~ 0.3	[126]

### Fully Tunable Bandgaps of Multinary TMDs

**Fig. 7:**
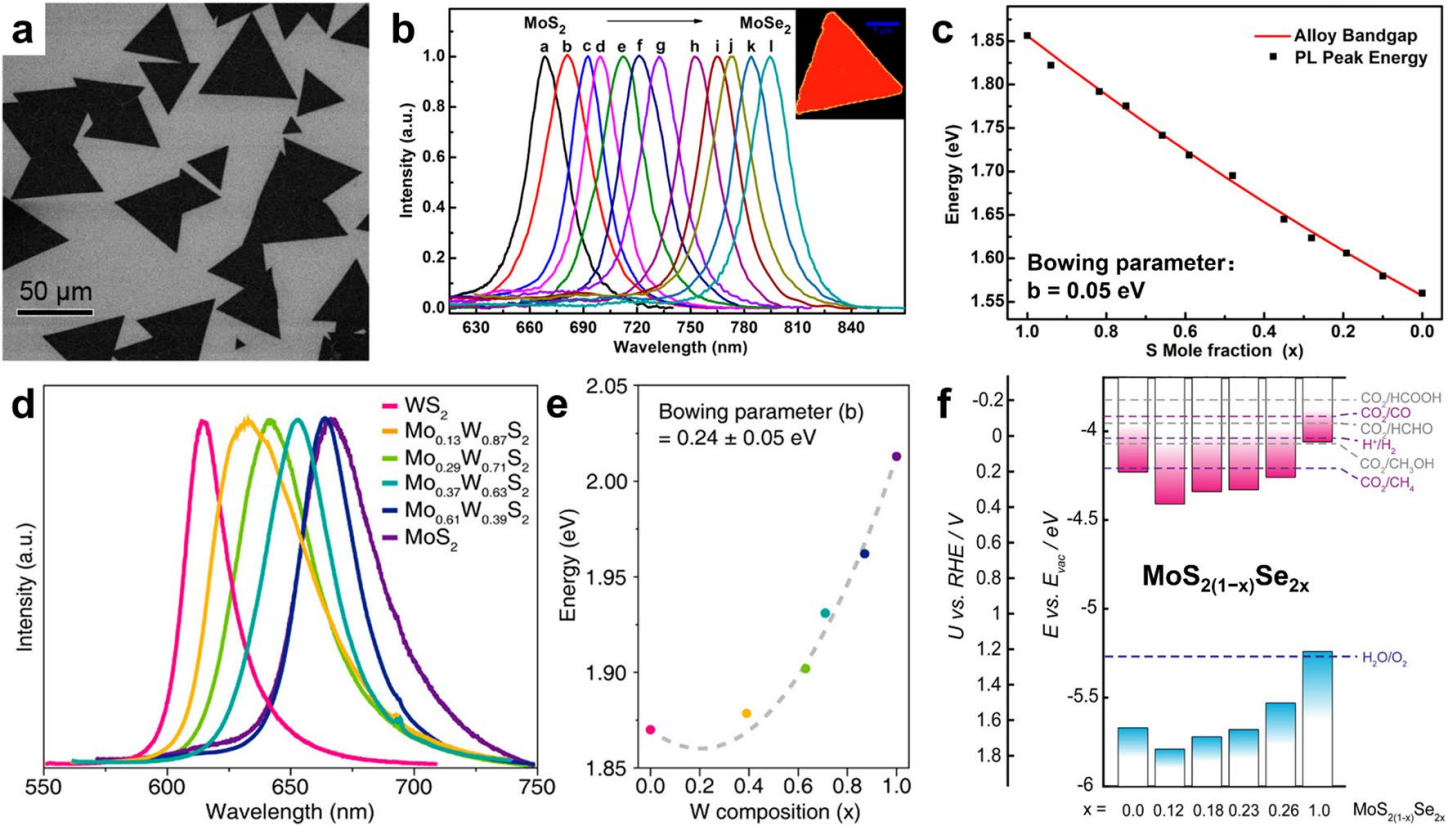
Composition-dependent band gap/edge in ATMDs. **a** Typical SEM morphology of the CVD-synthesized MoS_2*x*_Se_2(1−*x*)_ nanosheets. **b** PL spectrum of the MoS_2*x*_Se_2(1−*x*)_ nanosheets and a typical PL mapping of a single ternary nanosheet (inset) excited with a 488-nm laser. **c** Composition (x)-dependent bandgaps of the alloy nanosheets. Reproduced with permission from Ref. [44]. Copyright 2014 American Chemical Society. **d** PL spectra of Mo_1−*x*_W_*x*_S_2_ monolayers with the controlled compositions. **e** Measured optical bandgap of Mo_1−*x*_W_*x*_S_2_ monolayers (*x* = 0–1). Reproduced with permission from Ref. [131]. Copyright 2021, American Chemical Society. **f** Electronic band structures of few-layer MoS_2(1−*x*)_Se_2*x*_ films, with all energetic levels relative to the reduction potentials (vs. RHE) for aqueous CO_2_ reduction reaction (pH = 6.8). The valence band edges are derived by XPS, while optical band gaps are measured via ellipsometry. Reprinted with permission from Ref. [132]. Copyright 2020, American Chemical Society

Atomically substitutional engineering enables the alteration of bandgaps, band alignment/structure, and ultimately the optical and electronic properties of ATMD layers by exploiting the different electronic properties of multifarious TMDs. The introduction of an arbitrary number of heteroatoms can allow for fully tunable bandgap and tailored spectral response to be achieved across ATMD layers, making them ideal for next-generation electronic and optoelectronic devices. In 2014, Duan and his team synthesized high-quality and uniform MoS_2*x*_Se_2(1−*x*)_ nanosheets with fully tunable compositions via an improved CVD process [44]. The optical bandgap of MoS_2*x*_Se_2(1−*x*)_ can be continuously tuned from 1.557 eV (795 nm) to 1.856 eV (668 nm), as shown in Fig. 7a, b. This demonstrates the potential of atomically substitutional engineering for achieving tailored bandgap and spectral response in ATMD layers. The relationship between bandgap energy and composition x can be expressed as follows:

1 $${E}_{g}(x)=x{E}_{g}({{\text{MoS}}}_{2})+\left(1-x\right){E}_{g}({{\text{MoSe}}}_{2})-bx\left(1-x\right)$$

This equation, shown in Fig. 7c, describes the bandgap bowing effect in ATMDs, where the bowing parameter “b” is equal to 0.05 eV [44, 110, 128–130]. In another study, WS_2*x*_Se_2(1−*x*)_ nanosheets were fabricated to display a continuously tunable bandgap energy ranging from 1.649 eV (752 nm) to 1.979 eV (627 nm) [46]. Additionally, Mo_1−*x*_W_*x*_S_2_ alloy monolayers demonstrate composition-dependent bandgap and bowing effects, as shown in Fig. 7d, e. The bandgap is tunable from approximately 1.87 to 2.02 eV and has a larger bowing parameter of 0.24 ± 0.05 eV, serving as a typical example [131].

The differences in the bowing parameters of various ATMDs can be attributed to the diversity in electron filling of the d orbitals and lattice matching degrees between the two end-materials [41, 45, 66, 132–134]. MNX-type ATMDs, which involve two transition metal elements, generally have larger bowing parameters than MXY-type ATMDs [128, 129]. Additionally, the bandgap properties of ATMDs have been shown to be strongly dependent on layer number, temperature, and strain [135–137]. Interestingly, alloying of TMDs can modify their band edge positions (i.e., CBM and VBM), and the composition-dependent positions exhibit a similar bowing effect, as shown in Fig. 7f [132]. Furthermore, atomic substitution can alter the type of band alignment in material systems. For example, Zheng et al. developed PbI/WS_2(1−*x*)_Se_2*x*_ heterostructures via two-step CVD and discovered that increasing Se content in the alloys gradually alters their interfacial band alignments from type-I to type-II, accompanied by a change in PL emission from original enhancement (WS_2_) to final quenching (WSe_2_) [138]. In conclusion, atomically substitutional engineering can achieve a wide range and precise tuning of the bandgap and band alignment/structure, enabling the creation of novel optoelectronic and photovoltaic devices with tailored spectral response.

### Composition-dependent Raman Scattering of Multinary TMDs

Raman spectroscopy is a nondestructive and rapid tool used to investigate lattice vibrations and phonon dispersion of 2D nanomaterials. Researchers can use information such as peak frequency, peak intensity, and full width at half maxima (FWHM) in Raman spectroscopy to analyze the layer number, lattice orientation, strain, and other structural characteristics of 2D TMDs [139, 140]. Atomic substitution of TMDs changes their crystalline and electronic structures, which also alters their Raman scattering. As a result, researchers can creatively use Raman spectroscopy to characterize the composition and structure of ATMDs [139–141].

**Fig. 8:**
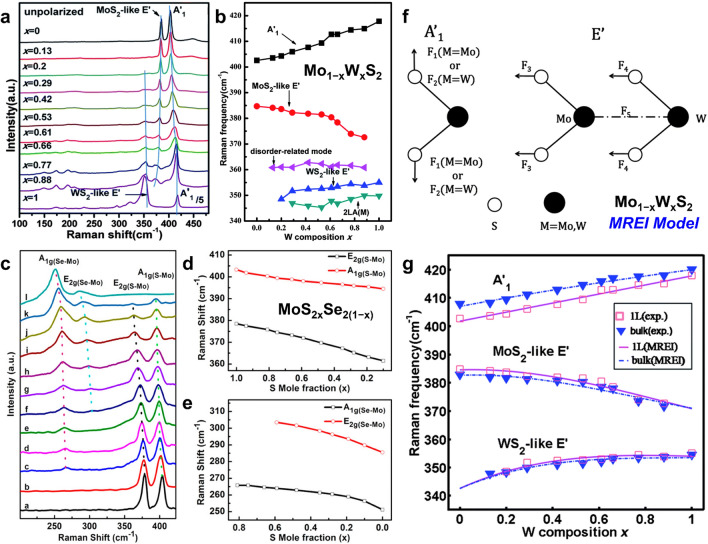
Composition-dependent Raman scattering in ATMD monolayers. **a** Raman spectra of Mo_1−*x*_W_*x*_S_2_ monolayers with different W compositions. The three blue lines show frequency shift of E’ and A_1_’ peaks with W composition. **b** Composition-dependent Raman frequencies of Mo_1−*x*_W_*x*_S_2_ monolayers. Reprinted with permission from Ref. [142]. Copyright 2014, Royal Society of Chemistry. **c** Raman spectrum of MoS_2*x*_Se_2(1−*x*)_ nanosheets excited with a 488-nm argon ion laser. **d–e** Raman shift of S–Mo related modes [E_2g(S–Mo)_, A_1g(S–Mo)_] and Se–Mo related modes [A_1g(Se–Mo)_, E_2g(Se–Mo)_] as S mole fraction. Reproduced with permission from Ref. [44]. Copyright 2014, American Chemical Society. **f** Schematic illustration of displacing atoms for the Raman active E’ and A_1_’ modes in Mo_1−*x*_W_*x*_S_2_ monolayer and force constants used in MREI model. **g** Composition-dependent Raman frequencies of E’ and A_1_’ (E_2g_^1^ and A_1g_ for bulk) modes in Mo_1−*x*_W_*x*_S_2_ alloys. The solid and dashed lines represent the fitting results of Mo_1−*x*_W_*x*_S_2_ monolayers and bulks via the MREI, respectively, while the scattered square and triangle points are the experimental data of Mo_1−*x*_W_*x*_S_2_ monolayers and bulks, respectively. Reprinted with permission from Ref. [142]. Copyright 2014, Royal Society of Chemistry

Studies have revealed that there are nine normal vibrational modes at the Γ point in the Brillouin zone for 2D (A)TMDs, among which the A_1_’ mode (out-of-plane) and E’ mode (in-plane) are Raman active [140]. For Mo_1−*x*_W_*x*_S_2_ monolayers, three characteristic peaks can be observed, assigned to the WS_2_-like E’, MoS_2_-like E’, and A_1_’ modes, respectively (Fig. 8a, b) [142]. Notably, the A_1_’ and E’ modes of Mo_1−*x*_W_*x*_S_2_ monolayers exhibit one-mode and two-mode composition-dependent behaviors, respectively. The FWHM of most peaks from Mo_1−*x*_W_*x*_S_2_ monolayers is larger than that of MoS_2_ and WS_2_ monolayers, indicating wider mode dispersion caused by well-blended and random alloys [142, 143]. In addition, a composition-insensitive disorder-related mode at ~ 360 cm^−1^ can only be observed in alloyed TMDs, implying the formation of disordered/random alloys instead of phase separation [142]. Similar behaviors have been found in other MNX-type ATMDs, such as Mo_1−*x*_W_*x*_Se_2_ and Mo_1−*x*_W_*x*_Te_2_ [49, 129, 144]. Distinctively, both the A_1_’ and E’ modes of most Re-based ATMDs (e.g., Mo_1−*x*_Re_*x*_S_2_, W_1−*x*_Re_*x*_S_2_) exhibit two-mode behavior, which is attributed to their tanglesome anisotropic crystal structure and the relatively large lattice mismatch between ReS_2_ and MoS_2_/WS_2_ [45, 124].

Figure 8c–e summarizes the Raman spectra and peak shift tendency of MoS_2*x*_Se_2(1−*x*)_ alloys with different S compositions [44]. The A_1g_ and E_2g_ peaks correspond to the branch of A_1_’ and E’ modes, respectively, and both modes exhibit two-mode behavior, as also demonstrated in WS_2*x*_Se_2(1−*x*)_ and HfS_2(1−*x*)_Se_2*x*_ alloys [46, 145]. It is worth noting that Se- and Te-based ATMDs often exhibit strong A_1_’ modes, making it challenging to observe the E’ characteristic peak, which can be studied using polarization Raman spectroscopy [46, 74, 108, 110, 142, 146, 147]. The atomic displacement for the E’ and A_1_’ modes in ATMDs, as illustrated in Fig. 8f, suggests that the E’ mode involves vibrations of both transition metal and chalcogen atoms, while the A_1_’ mode is only affected by chalcogen atom vibrations, revealing different behaviors in peak shift for different types of ATMDs [142]. These behaviors in Raman spectra imply the vibration and strain of the lattice in ATMDs, enhancing the surface activity of ATMD layers, ultimately enhancing the HER and gas-sensing performance [59, 127, 148].

The modified random-element-isodisplacement (MREI) model has been developed to simulate the lattice vibration behaviors of various TMD alloys. This model is based on two basal assumptions: (1) Atoms of the same kind vibrate with the same amplitude and phase, and (2) substituted atoms are randomly distributed [142, 149–151]. Through the MREI model, Xie’s group simulated the mode behaviors and fitted the composition-dependent Raman frequencies of A_1_’ and E’ modes in Mo_1−*x*_W_*x*_S_2_ monolayers, as outlined in Fig. 8f–g [142]. The fitting results are in good agreement with the experimental data, and the composition-dependent Raman frequency of the A_1_’ mode in Mo_1−*x*_W_*x*_S_2_ monolayer can be expressed as follows:

2 $${{\omega }_{{A}_{1}{\prime}}}^{2}=401.6\left(1+0.080x+0.002{x}^{2}\right)$$

By utilizing the MREI model combined with Raman spectroscopy, the aforementioned equation can be effectively employed to quantify the composition of Mo_1−*x*_W_*x*_S_2_ alloys.

## Improved Performances of Multinary TMDs

Atomically substitutional engineering allows for the creation of multinary TMDs with unique tunable properties such as a tunable bandgap, band alignment/structure, and crystal phase. These multinary TMDs often exhibit superior performance compared to binary TMDs. In this context, it is important to highlight the various advantages of multinary TMDs in a range of applications, including electronic and optoelectronic devices, as well as electrocatalysis, and more.

### Electronic Devices

Semiconducting binary TMDs have garnered widespread interest as field-effect transistors (FETs) due to their intriguing structural properties and exceptionally high electronic performance, including a high on/off ratio and carrier mobility in monolayer structures [152]. To further enhance the electrical performance of TMDs and explore their potential for nano-microdevices, heteroatom doping has been extensively studied to achieve *p*-/*n*-type modulation in TMDs [24, 25]. However, unlike conventional bulk 3D semiconductors, doping in atomically thin TMDs presents challenges in effectively modulating carrier density. The doping concentration in TMDs is often limited to less than 10% due to stringent lattice constant requirements, which obstructs the controllable synthesis and systematic investigation of doped TMDs [20, 28]. Interestingly, atomically substituted ternary TMDs not only inherit the unique layered structure of TMDs but also exhibit fascinating and full-composition-tunable electronic properties that can greatly improve their FET performance. This includes the ability to regulate carrier type, increase interface flexibility, and improve structural stability, which surpasses most hybridization strategies using external contacts [46, 58, 65, 66, 153].

#### Regulated Carriers in Type and Density

**Fig. 9:**
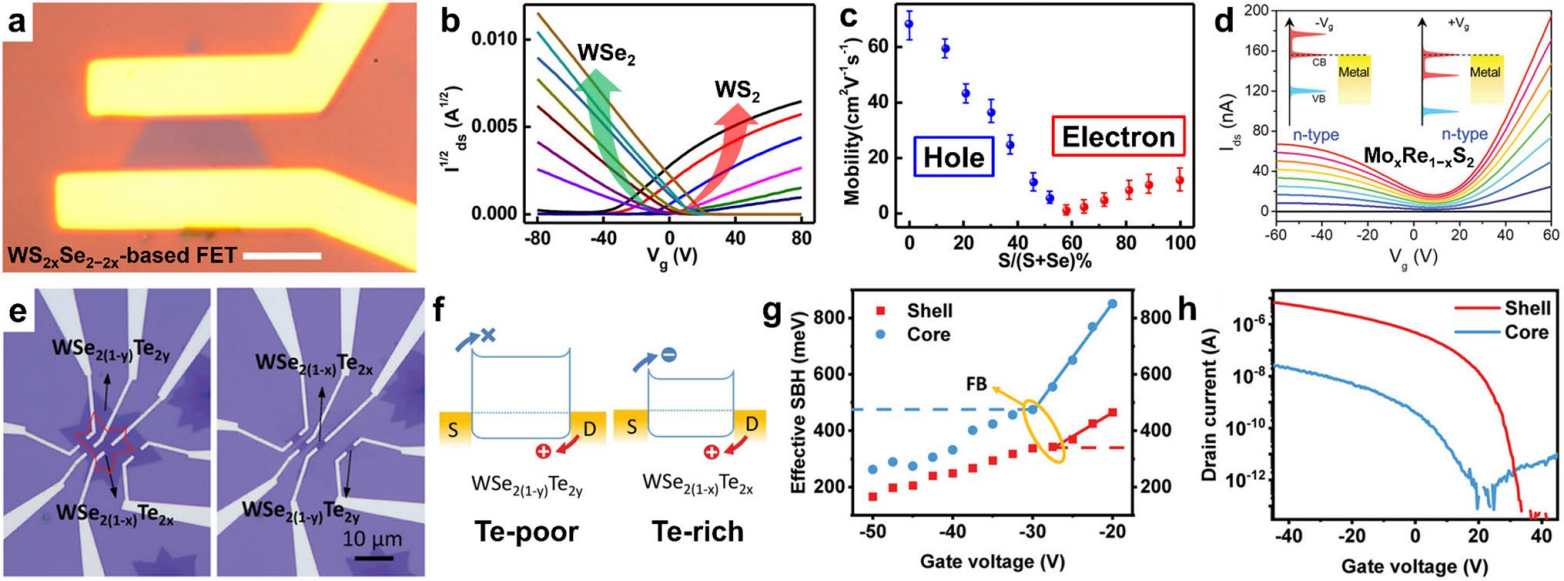
Composition-dependent electronic properties in ternary TMDs. **a–c** Electrical transport of WS_2*x*_Se_2−2*x*_ nanosheets: **a** optical microscopy of a typical back-gated field-effect transistor made of a WS_2*x*_Se_2−2*x*_ nanosheet (scale bar, 5 μm), **b** transfer characteristics (*I*_d_^1/2^–*V*_g_ plot) of WS_2*x*_Se_2−2*x*_ nanosheet transistors with different S atomic ratios from nearly pure WSe_2_ (brown curve) to nearly pure WS_2_ (black curve), and **c** field-effect mobility vs S atomic ratio in WS_2*x*_Se_2−2*x*_ alloy nanosheets, where the blue dots represent the hole mobility in WSe_2_-rich alloys and red dots represent electron mobility in WS_2_-rich alloys. Reproduced with permission from Ref. [46]. Copyright 2015, American Chemical Society. **d** Transfer curves (*I*_ds_–*V*_g_) of Mo_0.98_Re_0.02_S_2_ alloy FET, which presents a “bipolar-like” conduction behavior. Reproduced with permission from Ref. [45]. Copyright 2020, Wiley–VCH. **e–h** Electrical characterization of WSe_2−2*x*_Te_2*x*_/WSe_2−2*y*_Te_2*y*_ core/shell structure-based transistors: **e** optical image of the core/shell structure-based transistors. **f** Band alignments of core and shell transistors along the channel. The shell WSe_2−2*y*_Te_2*y*_ (Te poor) transistor has smaller hole Schottky barrier to facilitate hole transport, while the core WSe_2−2*x*_Te_2*x*_ (Te rich) has more balanced electron/hole Schottky barriers to favor ambipolar transport. **g** Effective Schottky barrier height as a function of gate voltage for shell and core transistors. **h** Electrical transport properties of the homogeneous transistors. The homogeneous shell transistor shows strong *p*-type transport, while the core transistor displays ambipolar behavior. The difference between the core and shell regions can be attributed to the bandgap difference between these two materials. Reproduced with permission from Ref. [157]. Copyright 2020, Wiley–VCH

Different binary TMDs generally exhibit different conductive types. For instance, MoS_2_ and WS_2_ grown on SiO_2_/Si substrates generally exhibit *n*-type conduction, while WSe_2_ exhibits *p*-type conduction [152, 154–156]. By alloying two kinds of TMDs with different conductive types, we can achieve fully adjustable instinctive electrical transport properties in a full range of compositions. Some Mo- and W-based ATMD layers exhibit a high degree of miscibility, resulting in their stability being even better than pristine TMDs [66]. As an example, Duan et al. fabricated WS_2*x*_Se_2−2*x*_ alloys with fully tunable compositions (*x* = 0–1) and carefully investigated their electrical transport properties through WS_2*x*_Se_2−2*x*_-based FETs with different compositions (Fig. 9a) [46]. As shown in Fig. 9b, Se-rich WS_2*x*_Se_2−2*x*_ alloys exhibit *p*-type conduction behavior, whereas S-rich WS_2*x*_Se_2−2*x*_ alloys demonstrate n-type conduction behavior. Additionally, weak bipolar behavior is observed in WS_2*x*_Se_2−2*x*_ alloys at the intermediate alloy region (0.4–0.65 S ratio). With an increase in the S ratio, the carrier types in WS_2*x*_Se_2−2*x*_-based transistors gradually change from hole to electron, indicating that the valence band and conduction band shift as the composition changes (Fig. 9c). Similar conduction behavior evolution has also been observed in ReS_2*x*_Se_2−2*x*_ and Mo_1−*x*_W_*x*_Se_2_ alloys [58, 130]. The tunable conduction behavior of ATMDs from *p*-type to bipolar and then to *n*-type can be attributed to the majority carriers of their two end-materials (i.e., *x* = 0 or 1) being holes and electrons, respectively, and their valence band or/and conduction band shifting as the alloy composition changes [41, 58, 132]. In another interesting study, the Mo_*x*_Re_1−*x*_S_2_-based FETs not only exhibited tunable conduction behavior dependent on composition but also had a novel “bipolar-like” electron behavior due to the formation of individual “sub-gap” in the conduction band. This could be used as a real bipolar FET to construct logic inverter devices (Fig. 9d) [45].

Yu et al. recently synthesized WSe_2(1−*x*)_Te_2*x*_ (*x* = 0–1) alloys that exhibit a metal–semiconductor phase transition, with Se-rich alloys demonstrating semiconducting 2H phase and Te-rich alloys showing metallic 1 T’ phase. Remarkably, Se-rich WSe_2(1−*x*)_Te_2*x*_-based FETs showed exceptional electronic performance with a maximum hole mobility of 46 cm^2^ V^−1^ s^−1^ and a high on/off ratio of 10^6^ at *x* = 0.3, while Te-rich WSe_2(1−*x*)_Te_2*x*_ exhibited metallic conductivity due to the rare Wyle semi-metallicity [48]. Moreover, monolayer alloys with a lateral core/shell structure, WSe_2−2*x*_Te_2*x*_/WSe_2−2*y*_Te_2*y*_ (*x* > *y*), were synthesized using a one-step CVD method with rapid cooling [157]. Differential band alignment was generated due to different Te concentrations in the core and shell regions, forming different Schottky barrier heights of electrons and holes in each region. This led to diverse conductive behaviors in the core and shell regions, with shell-based FETs (WSe_2−2*y*_Te_2*y*_, Te poor) exhibiting p-type conduction behavior with an excellent on/off ratio of 10^6^ and a high mobility of 30 cm^2^ V^−1^ s^−1^, while core-based FETs (WSe_2−2*x*_Te_2*x*_, Te rich) showed bipolar conductive behavior. The process is depicted in Fig. 9e–h. These findings demonstrate that ternary materials can exhibit different crystal phases and conductive properties at different compositions, providing a novel strategy for developing advanced exotic electronic devices with homo/heterojunctions. The current application of ternary TMDs in integrated electronic devices is at an early stage, primarily due to limitations caused by Coulomb impurity scattering resulting from the low dielectric constant of SiO_2_/Si substrates, as well as alloy scattering in ternary TMDs, which restricts carrier mobility. To overcome these limitations and enhance device performance, it is crucial to employ high-k dielectrics and optimize device structures [28, 46].

#### Enhanced Electronic Performances

**Fig. 10:**
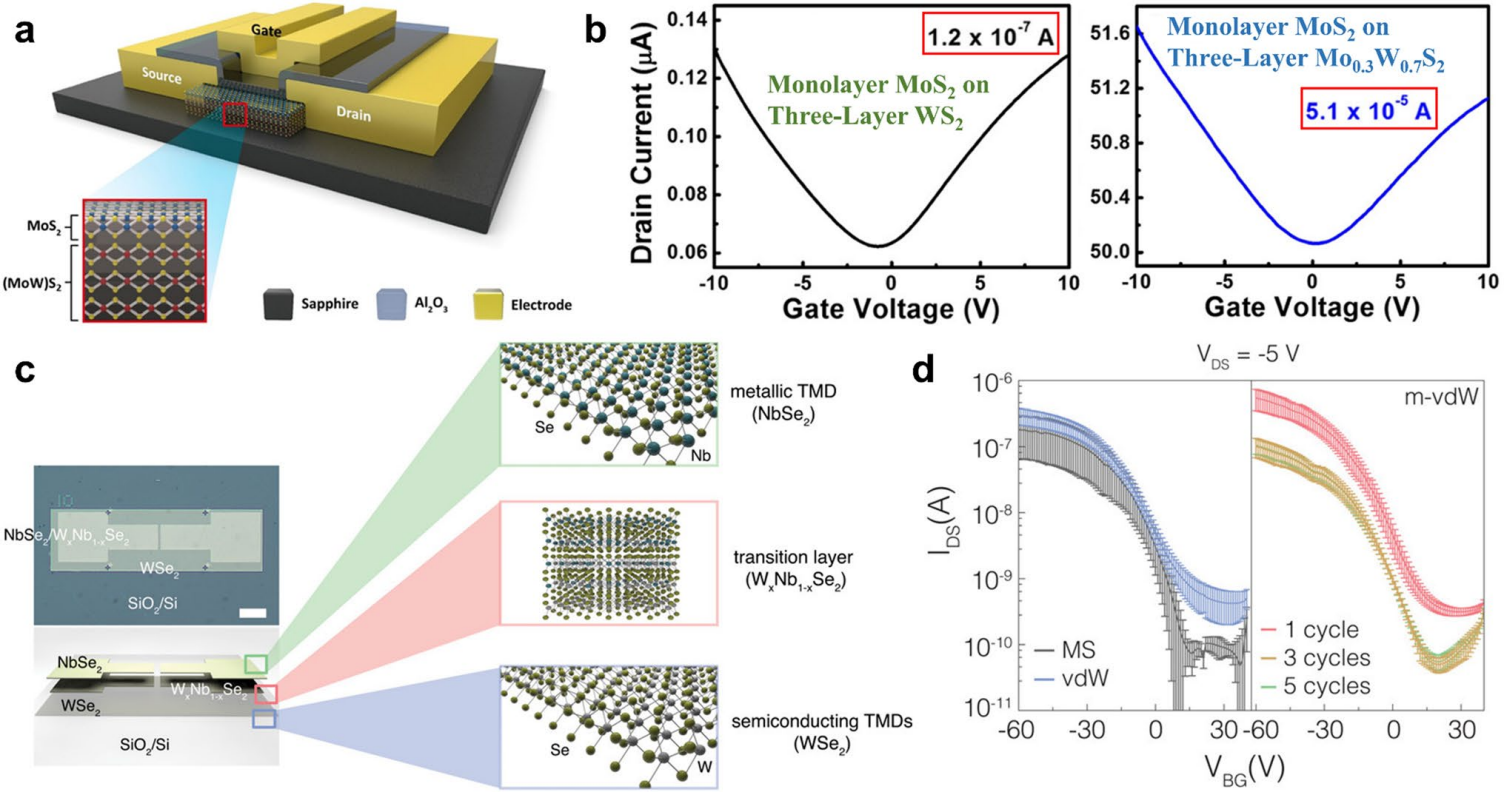
Improved device performance using ATMDs as interfaces. **a–b** Current enhancement and bipolar current modulation of top-gate transistors based on monolayer MoS_2_ on three-layer W_x_Mo_1−x_S_2_: **a** schematic illustration of the top-gate transistor. The gate electrode is separated from the conducting channel by an insulating layer of Al_2_O_3_. The panel shows the layer structure of the channel material, which is composed of monolayer MoS_2_ grown on Mo_*x*_W_1−*x*_S_2_. **b** *I*_D_–*V*_GS_ curves of the devices with monolayer MoS_2_ on three-layer WS_2_ (left) and Mo_0.3_W_0.7_S_2_ (right), respectively. The voltage *V*_DS_ was set to 2 V for all measurements. Reprinted with permission from Ref. [158]. Copyright 2018, American Chemical Society. **c–d** The interfacial transition region of W_*x*_Nb_1−*x*_Se_2_ for lowering the potential barrier height between the WSe_2_ channel and NbSe_2_ electrode in the WSe_2_-based bottom-gate FET with NbSe_2_ electrode: **c** optical image and schematic drawing of WSe_2_-based bottom-gate FET with NbSe_2_ electrode (scale bar, 100 μm). **d** Transfer characteristics (*I*_DS_–*V*_BG_) of FET devices with different electrode–channel structures: MS (metal–semiconductor, Pd–WSe_2_), vdW (NbSe_2_–WSe_2_), and m–vdW (mixed layer containing NbSe_2_–W_x_Nb_1−*x*_Se_2_–WSe_2_): 1, 3, and 5 cycle junction devices. Data were fitted by averages and standard deviations of 10 devices with each junction type. *I*_DS_ was measured at *V*_DS_ of − 5 V in devices with channel length of 50 μm and width of 100 μm. Reproduced with permission from Ref. [153]. Copyright 2016, American Chemical Society

Ternary TMDs have recently emerged as innovative van der Waals (vdW) interfacial layers for the fabrication of advanced electronic devices with enhanced performance. For example, Chen et al. developed top-gate transistors using monolayer MoS_2_ on three-layer Mo_*x*_W_1−*x*_S_2_ alloys, where enhanced electronic performance was achieved through atomic substitution-modified defect states and strains (Fig. 10a. b) [158]. Vu et al. employed a liquid-phase one-step CVD strategy to produce NbSe_2_/W_*x*_Nb_1−*x*_Se_2_ metal–semiconductor vdW heterostructures with precise control over atomic substitution ratios [159]. These vdW heterostructure-based FETs exhibited significantly superior electrical performance compared to pristine WSe_2_, including a raised carrier mobility of 27.24 cm^2^ V^−1^ s^−1^ (1,238 times improvement) and an on/off ratio of 2.2 × 10^7^ (4.400 times improvement), attributed to the substitution-modulated/optimized contacts and Schottky barrier heights between the channel and electrodes. In another study, Kim et al. designed a unique FET with semiconducting WSe_2_ channel and metallic NbSe_2_ electrodes, introducing an interfacial transition region (W_*x*_Nb_1−*x*_Se_2_) to boost charge transport between the metallic–semiconducting TMD interface and improve carrier mobility [153]. Compared to traditional FETs, this vdW stack structure-based FET showed increased charge injection efficiency and reduced Schottky barrier heights at the channel–electrode interfaces through quantum–mechanical tunneling (Fig. 10c, d). Therefore, optimizing contact resistance and Schottky barriers at the channel–electrode interfaces is important considerations in the design of ATMD-based FETs for advanced electronic devices with high performance.

### Optoelectronic Devices

#### Modulated Defect/Trap Levels

**Fig. 11:**
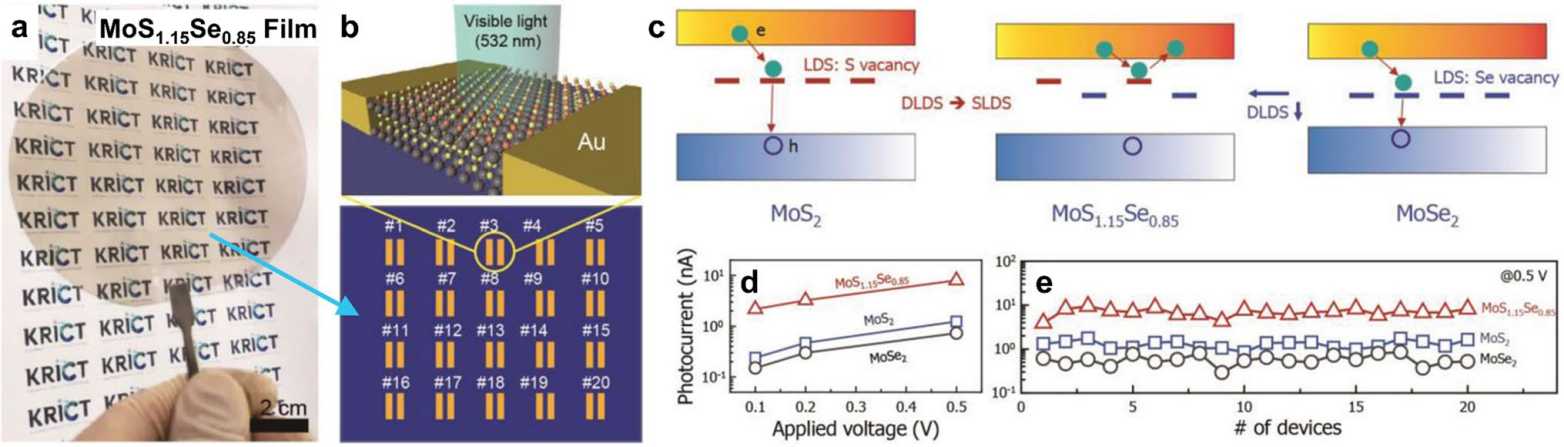
Enhanced photoelectronic performance of ternary TMDs over binary TMDs, making MoS_2(1−*x*)_Se_2*x*_ a promising candidate for use in industrial applications in nanophotonic devices. **a** Photograph of a large-scale, continuous multilayer MoS_1.15_Se_0.85_ film on a 4-inch quartz wafer. **b** Schematic illustration of MoS_1.15_Se_0.85_-based visible light photodetectors, including 20 devices with identical geometry. **c** Suggested band diagrams with localized defect states for MoS_2_, MoS_1.15_Se_0.85,_ and MoSe_2_. **d** Plots of photocurrent of MoS_2_, MoSe_2_, and MoS_1.15_Se_0.85_ as a function of bias voltage. **e** Device-to-device variations in photocurrent extracted from 20 devices for MoS_2_-, MoSe_2_-, and MoS_1.15_Se_0.85_-based photodetectors. Reprinted with permission from Ref. [163]. Copyright 2019, Wiley–VCH

Conventional binary TMDs often have high concentrations of chalcogen vacancies due to high-temperature synthesis, which severely limits their photoresponsivity and response time [116, 160–162]. To improve optoelectronic performance, atomic substitution of TMDs can be used to tailor their energy band structures and further to modulate their energy levels of defect state. In Lim’s work, a large-area and continuous uniform MoS_1.15_Se_0.85_ film was produced on a 4-inch quartz wafer and utilized to fabricate photodetectors (Fig. 11a, b) [163]. The band structure and defect states were modulated to optimize the absorption process for light and improve photoresponsivity. In particular, due to the band bowing effect caused by atomic substitution, the defect level of V_S_ (S vacancy) in MoS_1.15_Se_0.85_ is very close to its CBM, making it unlikely to be a recombination center and allowing captured electrons to be re-emitted into the conduction band as free carriers again (Fig. 11c). In other words, atomic substitution results in MoS_1.15_Se_0.85_ presenting a shallower energy level of defect state compared to MoS_2_ and MoSe_2_, making it emerge more photogenerated carriers. This transition of defect states from deep to shallow led to much-enhanced photoresponsivity compared to MoS_2_, MoSe_2_ (Fig. 11d), and other typical semiconductors (e.g., WS_2_, WSe_2_, Bi_2_Se_3_, and GaAs) [163]. Multiple devices with photoresponsive activity were used to characterize the uniformity of the alloy film, which is a significant step toward the industrialized manufacture and application of ATMD-based optoelectronic devices (Fig. 11e). Therefore, ternary TMDs hold great potential for improving optoelectronic performance by tailoring their energy band structures, which can pave the way for the development of advanced electronic devices.

The modulation mechanism for defect states in MNX-type TMDs can also be employed to enhance the photoresponsivity and accelerate the photoresponse rate. For instance, a high-performance photodetector was fabricated using PLD-grown Mo_0.5_W_0.5_S_2_ thin film with modulated band alignment and less defect concentration (i.e., S vacancy), which reduced photocarriers to be captured by the defect/trap states and shorten the response time to 150 ms [116]. Additionally, the relatively low defect concentration and shallower defect level enabled the Mo_0.5_W_0.5_S_2_-based photodetector to exhibit a superior responsivity of 5.8 A W^−1^ compared with pristine MoS_2_ and WS_2_ under *V*_ds_ of 2.2 V. Similarly, a ternary Mo_0.5_W_0.5_Se_2_-based photodetector was fabricated using the same synthetic method and physical mechanism, which showed much higher device performance compared to the binary MoSe_2_ and WSe_2_, including a photoresponsivity of 77.1 A W^−1^ and a response time of 8.3 ms [85]. Moreover, improved optoelectronic performance has been achieved in other ternary TMDs, such as Mo_1−*x*_Sn_*x*_S_2_ (a response time of 20 ms with comparison of 2.12 s for MoS_2_), WS_2(1−*x*)_Se_2*x*_ (a response time of 20 ms with comparison of 6.86 s for WS_2_ and 30 ms for WSe_2_), and ReS_2(1−*x*)_Se_2*x*_ (a response time of 15 ms with comparison of 60 ms for ReS_2_ and 20 ms for ReSe_2_), by suppressing deep-level defect states [52, 108, 109]. Overall, atomically substitutional engineering plays a significant role in modulating the energy levels of the defect/trap states and significantly improving the performance of ATMD-based optoelectronic devices.

The band engineering in ternary TMDs has inspired the fabrication of self-powered photodetectors using ternary TMDs with spatial composition gradients. For example, Xu et al. synthesized vertically stacked MoS_2(1−*x*)_Se_2*x*_ alloys with a spatially graded bandgap (induced by spatially graded composition *x*) using a liquid precursor-based CVD method, which exhibited a built-in electric field and can be utilized for self-powered photodetection [164]. In experiments, the self-powered phototransistors showed a sensitive gate-tuned photovoltaic effect and presented a photoresponsivity of 311 mA W^−1^ with an excellent detectivity of ~ 10^11^ Jones at 0 bias. Additionally, the biased devices delivered a remarkable photoresponsivity of 191.5 A W^−1^, a fast response time of 50 ms, and a photoconductive gain of 10^6^–10^7^ under light ranging from 405 to 808 nm, which demonstrated superior photoelectronic performance and photoresponse time in comparison with the photodetectors based on binary TMDs and even their heterostructures. This exciting work demonstrated promising prospects in structural design and bandgap engineering of 2D ATMDs for developing photoelectronic/photovoltaic devices.

#### Anisotropic and Broadening Photoresponse

**Fig. 12:**
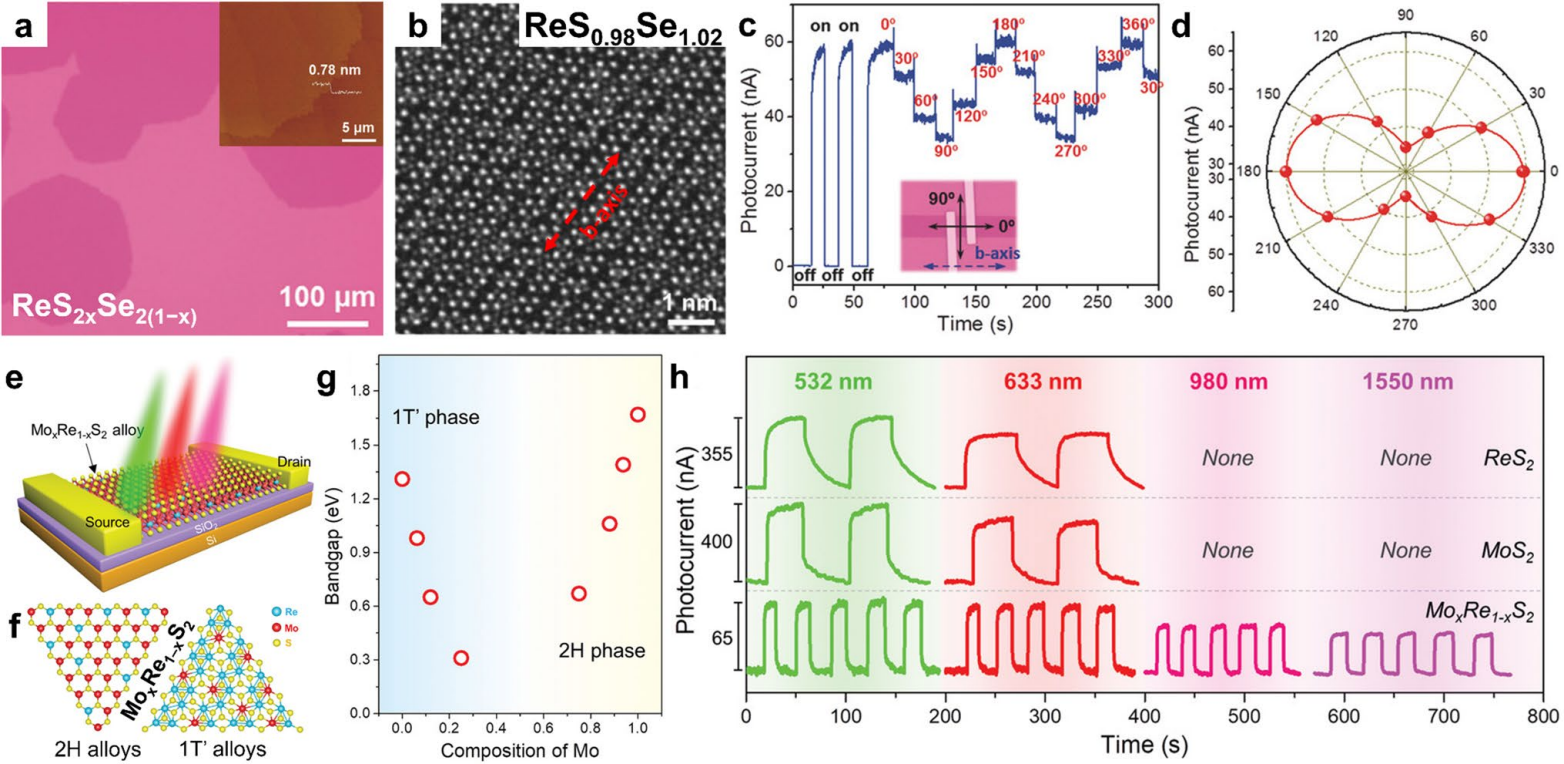
Anisotropic and broadened photoresponse. **a–d** Anisotropic photoresponse provided by ReS_2*x*_Se_2(1−*x*)_ alloys: **a** typical optical microscopy of monolayer ReS_2*x*_Se_2(1−*x*)_ alloys on SiO_2_/Si substrate and AFM image of as-grown ReS_2*x*_Se_2(1−*x*)_ alloys on mica substrate (inset). **b** ADF-STEM image of monolayer ReS_0.98_Se_1.02_ alloy. **c** Photocurrent response of ReS_1.06_Se_0.94_ device under light on and off irradiation, and the light with different polarization direction. The direction of b-axis is determined via ARPRS (angle-resolved polarized Raman spectra). **d** Polar plots for the photocurrent with respect to the polarization angle of the incident light. Reproduced with permission from Ref. [130]. Copyright 2017, Wiley–VCH. **e–h** Broadened photoresponse provided by Mo_*x*_Re_1−*x*_S_2_ alloys: **e** schematic diagram for the photoelectric measurement of the Mo_*x*_Re_1−*x*_S_2_ alloy device. **f** Atomic structure of 2H and 1T’ Mo_*x*_Re_1−*x*_S_2_ alloys. **g** Plots of bandgap as a function of the Mo composition x. **h** Plots of bandgap as a function of the Mo composition x. **i** Photocurrent as a function of time for ReS_2_ (the first row), MoS_2_ (the second row), and Mo_*x*_Re_1−*x*_S_2_ alloy (the third row) devices under light illumination with different wavelength: 532, 633, 980, and 1550 nm. The photoresponse measurements of these devices were taken at *V*_ds_ = 2 V, *V*_g_ = 0 V, the laser power density is ~ 5 mW mm^−2^. Reproduced with permission from Ref. [45]. Copyright 2020, Wiley–VCH

Re-involved ATMDs with anisotropic 1T’-phase crystal structures exhibit intriguing anisotropic photoresponses in optoelectronic devices, which depend on the polarization angle of the incident light [130, 165]. For example, in ReS_2*x*_Se_2(1−*x*)_-based optoelectronic devices, the photocurrent reaches the maximum and minimum values when the incident light is polarized along and perpendicular to the b-axis, respectively (Fig. 12a–d) [130]. Moreover, strong band bowing can be observed in Mo_*x*_Re_1−*x*_S_2_ alloys due to the large lattice mismatch and completely different crystal phase between MoS_2_ and ReS_2_, providing an exceedingly broadened spectral response range from visible to near-infrared light (Fig. 12e–h) [45]. Hence, Re-involved ATMDs play a vital role in designing futuristic anisotropy-based and specially tailored devices. Additionally, it was discovered that as the Te content in HfS_2(1−*x*)_Te_2*x*_ alloys increases from 0 (HfS_2_) to 0.19 (HfS_1.81_Te_0.19_), the bandgap decreases from 1.7 to 0.88 eV with significant band bowing. Consequently, the photoresponse wavelength of HfS_2(1−*x*)_Te_2*x*_-based photodetectors can be tremendously extended from visible to infrared light (1,310 nm) [166]. Benefiting from the strong band bowing effect, the specific atomic substitution in TMDs can greatly broaden their spectral response range, which provides a vital strategy for the development of high-performance TMD-based infrared photodetectors.

### Electrocatalytic Hydrogen Evolution Reaction

In today’s highly industrialized era, hydrogen is being considered as a potential sustainable and renewable substitute for fossil fuels. Electrocatalytic water splitting is one of the most effective and eco-friendly approaches for hydrogen production. Over the past decade, binary TMDs have been shown to be promising alternatives to noble metal-based electrocatalysts (such as Pt and Ir) for HER due to their unique layered structure, high specific surface area, and excellent stability [148]. However, the HER catalytically active sites in most binary TMDs are only located at the layer edges, with their basal planes being catalytically inert [148, 167, 168]. In contrast, ternary TMDs can introduce appropriate defects, induce lattice strain, facilitate phase transition, and even modify electronic structure to optimize the ΔG_H_, which greatly enhances the HER performance.

#### Enhanced Catalytic Activity by 2H-Phase Ternary TMDs

Gan et al. embedded Mo_*x*_W_1−*x*_S_2_ alloys onto flexible conductive carbon substrates (Mo_*x*_W_1−*x*_S_2_/C) to serve as HER electrocatalysts [143]. The local strain and grain boundaries induced by the alloying process resulted in a reduction of the energy barrier of hydrogen formation, leading to improved HER performance. In fact, the Mo_0.37_W_0.63_S_2_/C sample exhibited an impressively low overpotential of 0.137 V at j_geo_ = 10 mA cm^−2^ and a remarkable Tafel slope of 53 mV dec^−1^, better than that of the MoS_2_/C and WS_2_/C alternatives. Alternatively, CVD-grown WS_2(1−*x*)_Se_2*x*_ monolayers were also used as catalysts for HER. These monolayers presented a lower overpotential of 80 mV (j_geo_ = 10 mA cm^−2^) and a smaller Tafel slope of 85 mV dec^−1^ compared to WS_2_ and WSe_2_ [59]. The slight crystal distortion on the basal plane caused by lattice mismatch led to the formation of a polarized electric field in the plane, which contributed to the bond fracture of molecules adsorbed on the basal plane and thus enhanced the HER performance.

#### Enhanced Catalytic Activity by 1T/1T’-Phase Ternary TMDs

**Fig. 13:**
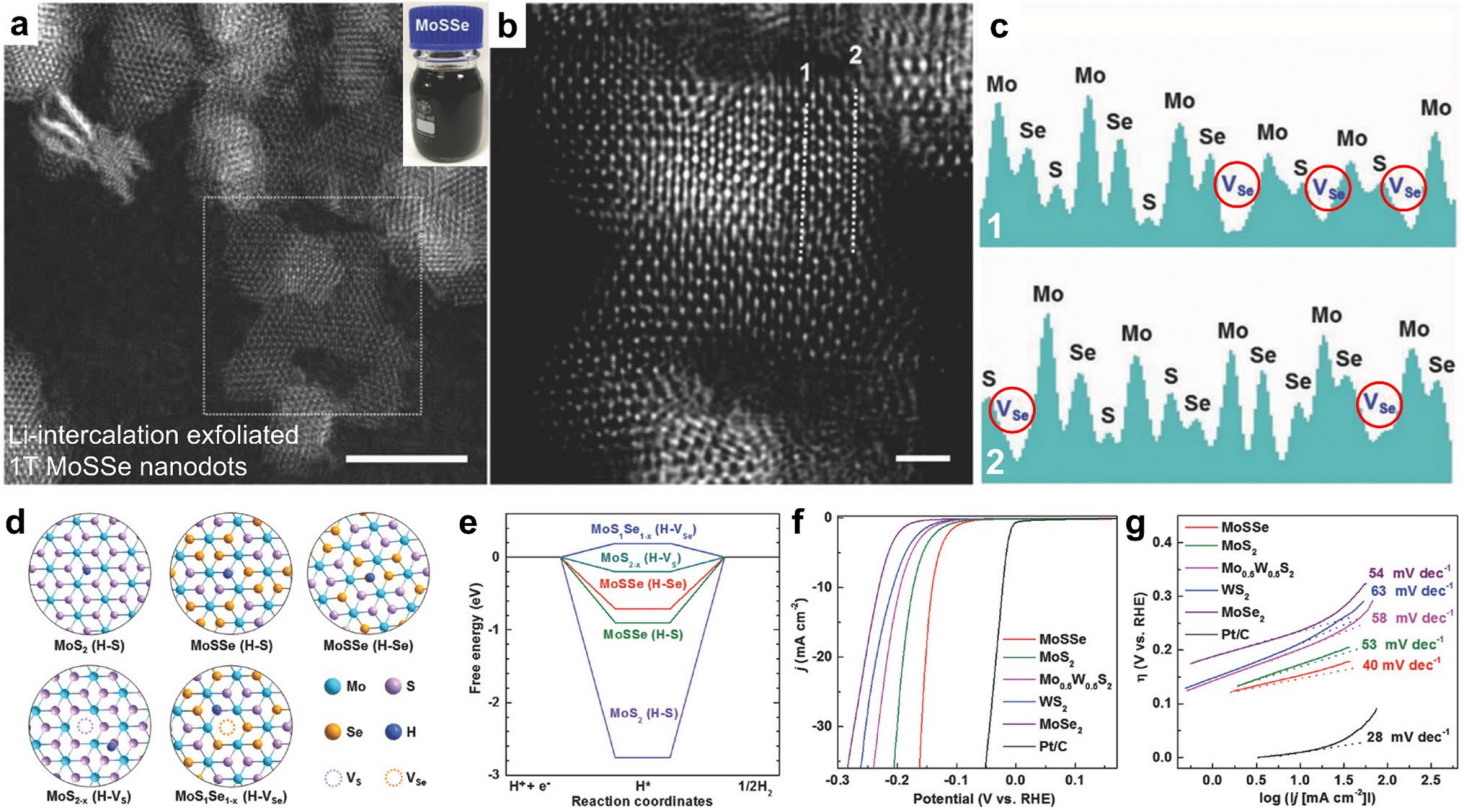
Enhanced HER performance provided by Li intercalation-exfoliated 1T-phase MoSSe nanodots. **a** Atomic resolution L2D-WF-filtered HAADF-STEM of MoSSe nanodots. Inset in **a**: photograph of aqueous solution of MoSSe nanodots (scale bar, 5 nm). **b** Enlarged L2D-WF-filtered image of MoSSe nanodots shown in the dotted square in **a** (scale bar, 1 nm). **c** Brightness profiles along the dotted lines in **b**. V_Se_ in the brightness profiles represents the Se vacancy. **d** Atomic models for hydrogen atoms adsorbing at the active sites of basal planes of 1 T-phase MoS_2_, 1T-phase MoSSe, 1T-phase MoS_2_ with S vacancy, and 1T-phase MoSSe with Se vacancy. **e** Calculated free energy versus the reaction coordinates of HER for the basal planes of various catalysts. **f** iR-corrected polarization curves of Li intercalation-exfoliated 1T-phase MoSSe, MoS_2_, Mo_0.5_W_0.5_S_2_, WS_2_, and MoSe_2_ nanodots, and the commercial Pt/C catalysts. **g** The corresponding Tafel slopes of these catalysts derived from **f**. Reproduced with permission from Ref. [53]. Copyright 2018, Wiley–VCH

Research has shown that ternary TMDs with 1 T/1 T’ phase are more advantageous for HER than the 2H phase, due to their comparatively high-density exposed active sites and metallic conductivity [148]. Zhang et al. employed a ball milling and Li-ion intercalation method to synthesize high-percentage metallic 1T-phase MoSSe nanodots from corresponding 2H-phase bulk crystals [53]. These nanodots exhibited high-density catalytically active edge sites and Se vacancies (Fig. 13a–c), and first-principle calculations revealed that alloying of 2D TMDs reduces the Gibbs free energy for atomic hydrogen adsorption (Δ*G*_H_). Furthermore, experimental characterization confirmed that the Se vacancy (V_Se_) in MoSSe nanodots further reduces the Δ*G*_H_ to enhance HER catalytic activity (Fig. 13d, e). The combined effect of high-density exposed catalytic active sites, high conductivity of metallic 1T phase, and the reduced Δ*G*_H_ led to significantly improved HER performance compared to MoS_2_ and MoSe_2_ nanodots (Fig. 13f, g). Similarly, ReSSe nanodots with intrinsic distorted 1T’ structure were fabricated and demonstrated greatly modified HER performance compared to ReS_2_ and ReSe_2_ nanodots due to the alloying effect and the asymmetric S vacancies [169].

**Fig. 14:**
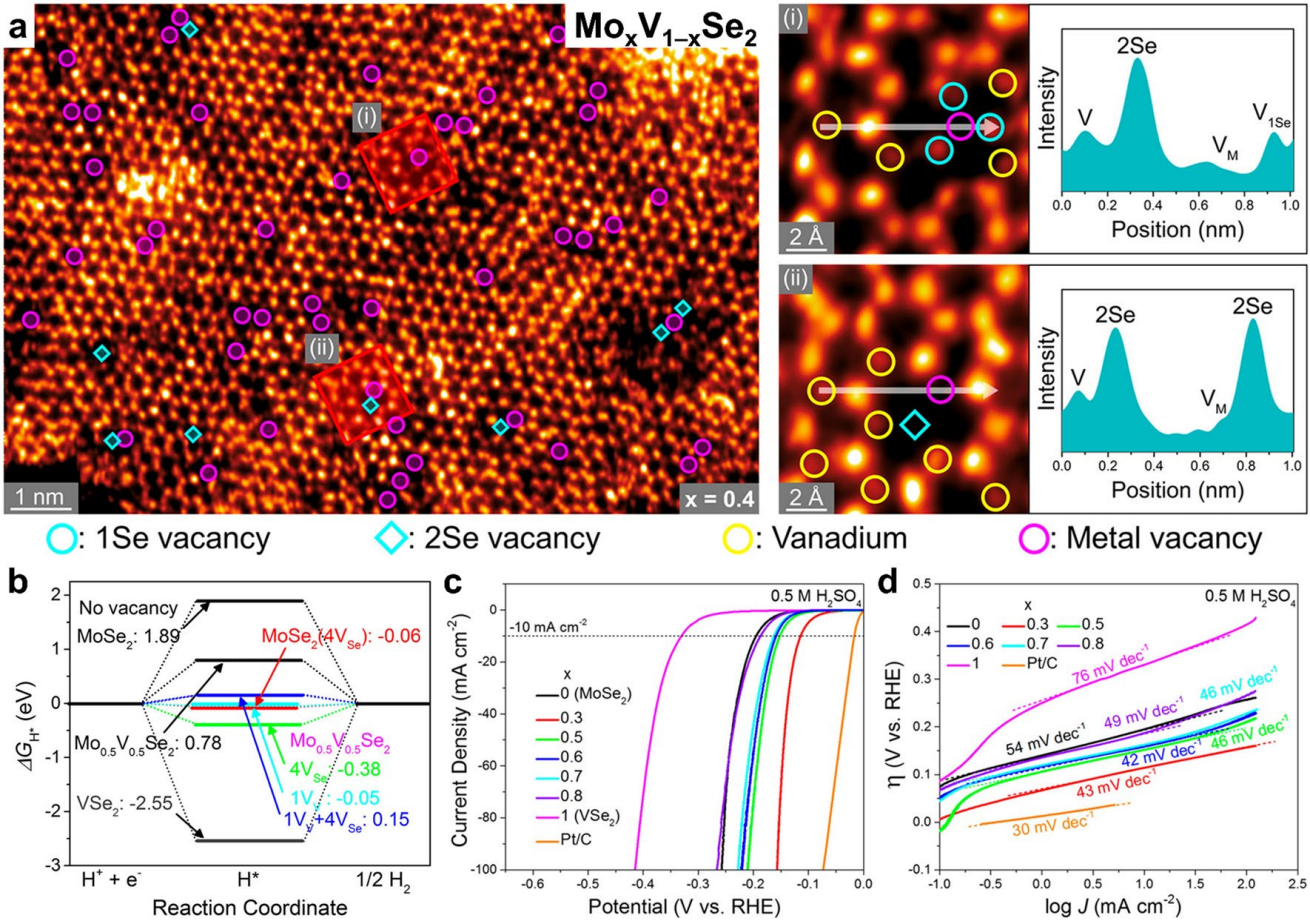
Enhanced HER performance provided by vacancy-rich Mo_1−*x*_V_*x*_Se_2_ nanosheets. **a** Atomically resolved HAADF-STEM images of vacancy-rich Mo_0.6_V_0.4_Se_2_ (with intensity profile along the white line). The darker regions contain the V vacancies (marked by magenta circles). The V atom sites are labeled by yellow circles, and the Se vacancies (1Se: monovacancy, 2Se: divacancy) are marked by cyan circles and diamonds, respectively. In the line profiles, V_M_ and V_1Se_ represent the metal vacancy and Se monovacancy sites, respectively. **b** Gibbs free energies (Δ*G*_H*_) for *x* = 0, 0.5, and 1 with different vacancy structures. **c** LSV curves with scan rate of 2 mV s^−1^ for Mo_1−*x*_V_*x*_Se_2_ and Pt/C catalysts in 0.5 M H_2_SO_4_. **d** Tafel plots derived from the LSV data measured at low potential region. Reproduced with permission from Ref. [54]. Copyright 2021, American Chemical Society

In addition to the 1T/1T’ phase induced by Li-ion intercalation and the inherent one in binary TMDs, the substitution-induced phase transition from 2H to 1T/1T’ also exhibits improved performances in HER application. Kang et al. synthesized Mo_1−*x*_V_*x*_Se_2_ nanosheets with fully tunable composition (*x* = 0–1) via a colloidal reaction, which showed a tunable phase from 2H to 1T with increasing *x* [54]. The two end-materials possess completely different crystalline phases (i.e., 2H phase for MoSe_2_, 1T-phase for VSe_2_), leading to the induction of a mass of multifarious atomic vacancies (such as Se monovacancy, Se divacancy, V vacancy) in their ternary nanosheets. These abundant vacancies act as catalytically active sites for the HER reaction (Fig. 14a). Computational studies verified that alloying can significantly reduce the formation energy of atomic vacancies, and both the alloying itself and the atomic vacancies can reduce Δ*G*_H_ to enhance the catalytic activity of HER (Fig. 14b). The HER performance of Mo_1−*x*_V_*x*_Se_2_ alloys with different compositions was systematically investigated in Kang’s work, and it was found that the best performance was achieved at *x* = 0.3 due to the existence of the most abundant atomic vacancies (Fig. 14c, d). The same research group performed another important work to produce Mo_1−*x*_Nb_*x*_Se_2_ (*x* = 0–1) ternary nanosheets with varying compositions in a full range through a solvothermal reaction [93]. These nanosheets had large lattice mismatches and were comprehensively investigated for their potential in HER. HAADF-STEM revealed the presence of diverse atomic defects, including Se and Nb vacancies, concurrent vacancies, and even adatom defects. DFT calculations confirmed that the ternary nanosheets had obviously lower formation energies for vacancies compared to the binary counterparts. As a consequence, abundant defect sites and substitution-reduced Δ*G*_H_ synergistically enhanced the HER performance of the ternary TMD nanosheets. Furthermore, more phase transition alloy nanosheets, including Re_1−*x*_Mo_*x*_S_2_, Re_1−*x*_Mo_*x*_Se_2_, W_1−*x*_V_*x*_Se_2_, and Nb_1−*x*_V_*x*_Se_2_, were fabricated to exploit the best HER performance by the alloying formation of 1T/1T’ phase and introduction of atomic vacancies [55, 94, 121, 126]. The enhanced HER performance provided by a variety of ternary TMDs is summarized in Table 4.

**Table 4:** Comparison of HER performance of diverse TMDs with different morphologies and crystalline phases with their corresponding end-materials

2D (A)TMDs	Synthetic methods	Morphology	Phase	Tafel slope	Overpotential	References
MoS_0.98_Se_1.02_	CVD	Monolayer films	2H	119	273	[134]
MoS_2_			2H	134	335	
MoSe_2_			2H	134	303	
MoSSe	Li-ion intercalation	Nanodots	1T	40	140	[53]
MoS_2_			1T	53	173	
MoSe_2_			1T	54	209	
ReSSe	Li-ion intercalation	Nanodots	1T’	50.1	84	[169]
ReS_2_			1T’	106.9	320	
ReSe_2_			1T’	50.8	123	
WS_1.14_Se_0.86_	CVD	Triangular monolayer nanosheets	2H	85	80	[59]
WS_2_			2H	95	100	
WSe_2_			2H	100	150	
rGO/W_0.4_Mo_0.6_S_2_	Wet chemical method	Heterojunction thin films	2H	38.7	96	[170]
rGO/WS_2_			2H	68.4	150	
rGO/MoS_2_			2H	82.0	197	
Mo_0.37_W_0.63_S_2_/C	CVD	Membranes	2H	53	137	[143]
MoS_2_/C			2H	68	178	
WS_2_/C			2H	60	166	
Re_0.55_Mo_0.45_S_2_	CVD	Monolayer flakes	1T’	56	147	[120]
ReS_2_			1T’	200	438	
MoS_2_			2H	134	475	
MoS_2_			1T	77	216	
Re_0.9_Mo_0.1_Se_2_	Hydrothermal reaction	Nanosheets	1T’	42	77	[55]
ReSe_2_			1T’	61	107	
MoSe_2_			2H	77	188	
Mo_0.5_Nb_0.5_Se_2_	Solvothermal reaction	Nanosheets	2H	46	140	[93]
MoSe_2_			2H	56	207	
NbSe_2_			2H	85	292	
Mo_0.7_V_0.3_Se_2_	Colloidal reaction	Nanosheets	2H	43	114	[54]
MoSe_2_			2H	54	195	
VSe_2_			1T	76	330	
Nb_0.7_V_0.3_Se_2_	Colloidal reaction	Nanosheets	2H/1T	72	236	[126]
NbSe_2_			2H	86	295	
VSe_2_			1T	107	386	
W_0.9_V_0.1_Se_2_	Colloidal reaction	Nanosheets	2H	80	128	[121]
WSe_2_			2H	83	168	
VSe_2_			1T	108	387	

The Tafel slope is measured in mV dec^−1^ and the overpotential is measured in mV at *j* = 10 mA cm^−2^

### Other Applications

**Fig. 15:**
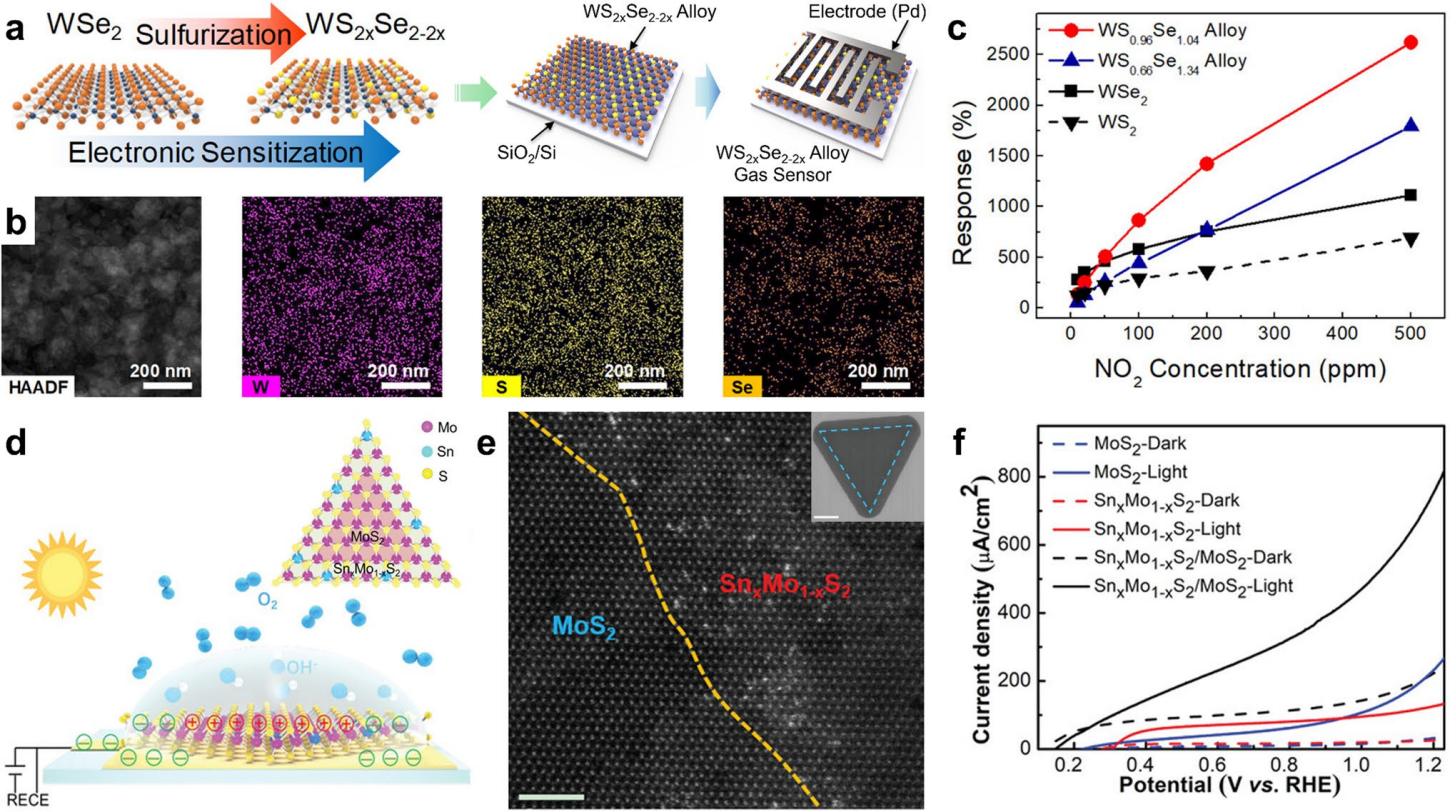
**a–c** Enhanced gas-sensing performance provided by WS_2*x*_Se_2−2*x*_ layers: **a** schematic for the fabrication process of the WS_2*x*_Se_2−2*x*_ alloy-based gas sensors. **b** HAADF TEM image of WS_0.96_Se_1.04_ alloy and EDS elemental maps of W, S, and Se in HAADF images. **c** Response of WSe_2_, WS_0.66_Se_1.34_ alloy, WS_0.96_Se_1.04_ alloy, and WS_2_ gas sensor for NO_2_ exposure as a function of gas concentration. Reproduced with permission from Ref. [127]. Copyright 2018, American Chemical Society. **d–e** Enhanced photoelectrocatalytic performance provided by Sn_*x*_Mo_1−*x*_S_2_/MoS_2_ heterostructure as a photoanode: **d** schematic illustration of the photoelectrocatalysis measurements. The Sn_*x*_Mo_1−*x*_S_2_/MoS_2_-on-quartz sample was connected in the external circuit with Ti electrodes. All the measurements were taken in 0.5 M Na_2_SO_4_ solutions. The light illumination was produced using a 300-W Xe lamp unless otherwise specified. Inset in **d**: atomic structure diagram of the lateral Sn_*x*_Mo_1−*x*_S_2_/MoS_2_ heterostructure. **e** Atomic resolution STEM image taken from the epitaxial metal–semiconductor heterostructure at the interface. The yellow dotted line indicates the atomic interface. Inset is a typical SEM image of Sn_*x*_Mo_1−*x*_S_2_/MoS_2_ heterostructure. **f** Linear-sweep voltammogram curves of different anodes. Solid line: light irradiation. Dashed line: dark. Reprinted under the terms of the Creative Commons Attribution License from Ref. [172]. Copyright 2020, The Authors, published by Wiley–VCH

In addition to their achievements in various applications including HER, ternary TMDs are promising materials for gas-sensing and photoelectrocatalysis, although related research is in its early stages. For example, Ko et al. fabricated large-area and uniform WS_2*x*_Se_2−2*x*_ alloy films via a sulfurization process based on CVD (Fig. 15a, b) [127]. Through electronic sensitization and surface modification, the WS_0.96_Se_1.04_-based gas sensor exhibited a 2.4 times enhanced response compared to WSe_2_ when exposed to 500 ppm NO_2_ (Fig. 15c). Furthermore, the WS_0.96_Se_1.04_-based gas sensor demonstrated good flexibility and outstanding long-term stability. In Xu’s work, MoSeS alloy monolayers were produced by a novel liquid–liquid interface-mediated strategy and employed in electroreduction of CO_2_ into syngas (i.e., CO and H_2_) [171]. Remarkably, the MoSeS monolayers showed a current density of 43 mA cm^−2^ at − 1.15 V vs. RHE, which was 2.7 and 1.3 times higher than that of MoS_2_ and MoSe_2_ monolayers, respectively. Moreover, the MoSeS monolayers exhibited a much higher Faradaic efficiency (FE) of 45.2% for CO production at − 1.15 V compared to MoS_2_ (16.6%) and MoSe_2_ (30.5%) monolayers. DFT calculations indicated that the eccentric charge around Mo atoms in MoSeS alloys not only stabilizes the COOH* intermediate but also promotes the rate-limiting step of CO desorption, greatly enhancing electrocatalytic performance. More recently, Shao et al. fabricated lateral epitaxial metal–semiconductor Sn_*x*_Mo_1−*x*_S_2_/MoS_2_ heterostructures via a one-step CVD method, in which the edge of MoS_2_ is modified with Sn atoms in situ to form metallic Sn_*x*_Mo_1−*x*_S_2_ alloys, resulting in seamless splicing of metallic Sn_*x*_Mo_1−*x*_S_2_ at semiconducting MoS_2_ edge (Fig. 15d, e) [172]. As a result, due to efficient electron–hole separation and fast charge transfer, the heterostructures showed improved performance in photoelectrocatalytic oxygen evolution, including a lower onset potential of 0.15 V and a higher current density of > 0.8 mA cm^−2^ at 1.23 V vs. RHE, which was 2.5 times higher than that of pristine MoS_2_ (Fig. 15f).

## Emerging Janus Ternary TMD Monolayers

Similar to the gold Janus in Roman mythology, a unique type of MXY-type ATMD layer can be found, where a chalcogen element (X) is present on the upper side of the transition metal, while another chalcogen element (Y) is located on the lower side of the transition metal. The highly ordered ternary structure of Janus TMD monolayers (JTMDs) breaks the out-of-plane symmetry, generating spontaneous vertical dipole moments [50, 60]. Since the fabrication of Janus SMoSe in 2017, JTMDs have received considerable attention, and their fascinating and unique properties have been predicted theoretically and investigated experimentally, such as Zeeman- and Rashba-type spin splitting, rapid exciton formation, tribological piezoelectricity, intrinsic ferroelasticity, and large valley polarization [50, 60, 173–177].

**Fig. 16:**
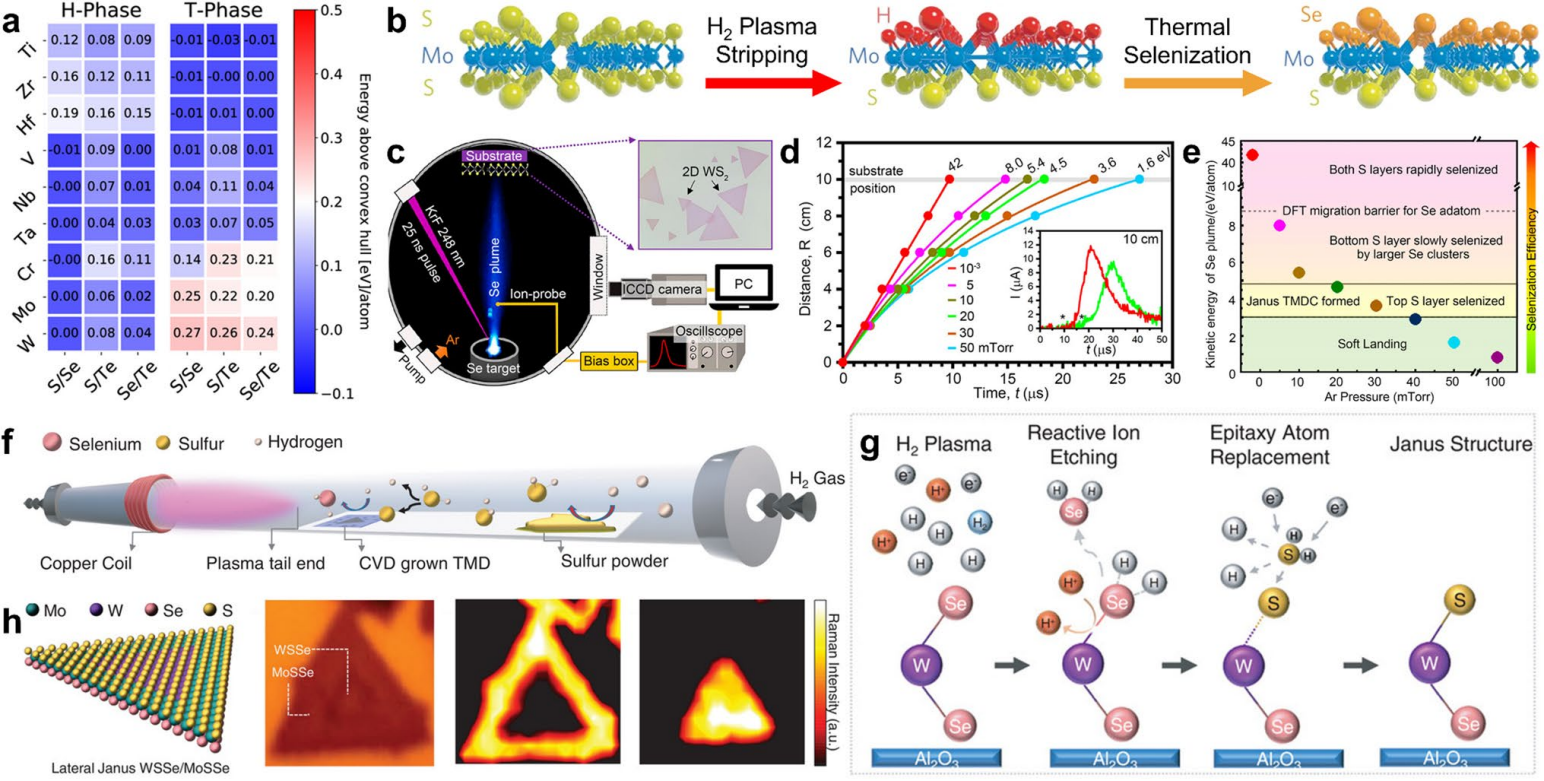
Strategic preparation of Janus TMDs. **a** Thermodynamic stability of the H-phase and T-phase for different Janus XMY monolayers. The colors denote the energy above the convex hull in eV/atom. Reprinted with permission from Ref. [178]. Copyright 2019, American Chemical Society. **b** Synthesis of the Janus SMoSe monolayer. A MoS_2_ monolayer grown by CVD is exposed to H_2_ plasma to strip the top-layer S. The plasma is then switched off, and a quartz boat loaded with Se powder is moved next to the SMoH sample without breaking the vacuum. Se powders are then thermally vaporized to achieve selenization and complete the synthesis of Janus SMoSe monolayers. Reprinted with permission from Ref. [50]. Copyright 2017, Nature Publishing Group. **c–e** PLD-based low-energy implantation into 2D TMDs to form Janus structures: **c** experimental setup for Se plasma generation and impingement on CVD-grown WS_2_ monolayer within a vacuum chamber equipped with an ICCD camera and a translatable probe for ion-flux measurement. **d** *R–t* plots of the leading edge of the plasma (from ion probe currents, see * in inset) track the propagation and deceleration in different background Ar pressures. **e** Summary diagram of KE regimes for selenization of WS_2_ monolayer by using Se PLD. Selenization of only the top S layer of WS_2_ monolayer suitable for Janus SWSe formation occurs between 20 and 40 mTorr for Se plume KEs between 3 and 4.5 eV atom^−1^. At low pressures (≤ 20 mTorr) and plume KEs above 5.4 eV atom^−1^, selenization of the bottom S layer by larger Se clusters increases and completes rapidly once pressures decrease toward vacuum. Reproduced with permission from Ref. [182]. Copyright 2020, American Chemical Society. **f–h** Room-temperature synthesis of 2D Janus TMDs. **f** Schematic demonstration of the selective epitaxy atomic replacement (SEAR) process through inductively coupled plasma for the synthesis of 2D Janus TMDs. **g** Working scheme of room-temperature SEAR process. **h** The atomic representation and optical image of Janus SMoSe/SWSe lateral heterostructures, and Raman mapping at 290 and 284 cm^−1^ for characteristic Janus SMoSe and SWSe peaks. Reproduced with permission from Ref. [101]. Copyright 2020, Wiley–VCH

***Approaches for Synthesizing Janus Ternary TMDs:*** Researchers from Thygesen’s group evaluated the thermodynamic stability of 216 Janus monolayers in both H-phase and T-phase using calculated energy above the convex hull (*E*_hull_), which represents the energy of the most stable phase (possibly mixed) as a function of stoichiometry [178]. As shown in Fig. 16a, the H-phase JTMDs of Group VB or VIB transition metals and T-phase JTMDs of Group IVB or VB transition metals have relatively high thermodynamic stability and are expected to be successfully synthesized. Additionally, the researchers assessed the dynamic stability of all 216 Janus monolayers and computed the magnetic, elastic, electronic, and optical properties of the 70 most stable Janus monolayers. These results were uploaded to the Computational 2D Materials Database, providing a powerful guide for the fabrication and preservation of Janus monolayers [179, 180]. Moreover, the atomically thin nature of Janus XMY monolayers endows their (opto)electronic properties highly sensitive to various structural defects. Therefore, it is crucial to have a comprehensive understanding of the formation and influences of different types of defects on the (opto)electronic properties of Janus monolayers before their fabrication and application. Zhang et al. conducted first-principles calculations to evaluate the thermodynamic stability of different defects in Janus SMoSe and their impact on its electronic properties [181]. The results revealed that point vacancies of S and Se atoms were the most thermodynamically favorable compared to line defects and 60° grain boundaries. Additionally, polar-discontinuous 60° grain boundaries tend to exhibit characteristics of 1D metallic quantum wires.

Due to its unique structure, the synthesis of JTMDs requires precise reaction conditions. Currently, JTMDs are fabricated using CVD-grown binary TMD monolayers [50, 51, 101, 182, 183]. The simplest method involves sulfurizing CVD-grown MoSe_2_ at high temperatures to produce Janus SMoSe [51]. An alternative method involves stripping the top-layer S atoms of CVD-grown MoS_2_ and replacing them with H atoms via hydrogen plasma treatment, followed by selenization of the intermediate Janus SMoH to form Janus SMoSe (Fig. 16b) [50, 183]. In both methods, the temperature and carrier gas flow rate must be precisely controlled to prevent further sulfurization/selenization or the formation of ATMDs with random atomic arrangements. To simplify the process, Lin and his colleagues developed a pulsed laser deposition (PLD)-based strategy to form Janus SWSe by implanting Se clusters into CVD-grown WS_2_ at 300 °C (Fig. 16c) [182]. The kinetic energy (KE) of Se species can be controlled by adjusting the background Ar pressure, which allows for the controlled conversion of WS_2_ into Janus SWSe, WS_2(1−*x*)_Se_2*x*_ random alloys, and even WSe_2_ by precisely altering the KE during the process of repeated Se implantation and recrystallization (Fig. 16d, e). Recently, Trivedi et al. demonstrated a selective epitaxy atomic replacement (SEAR) method for preparing JTMDs and their heterostructures at room temperature (Fig. 16f–h) [101]. Janus SWSe was used as an example, where the H radicals combined with the Se atoms at the top of CVD-grown WSe_2_ to form intermediates that were then bombarded and split with high-energy H^+^ ions to generate Se vacancies. The introduction of sulfur during the SEAR process is essential to fabricate Janus SWSe, where H radicals react with sulfur to form H_2_S vapor that is transported toward the samples by the carrier gas and dissociated into S and H radicals. Ultimately, the S radicals incorporate into chemically active Se vacancies to form stable Janus SWSe (Fig. 16g). Importantly, this process is performed at room temperature and driven by kinetics rather than thermodynamics.

**Fig. 17:**
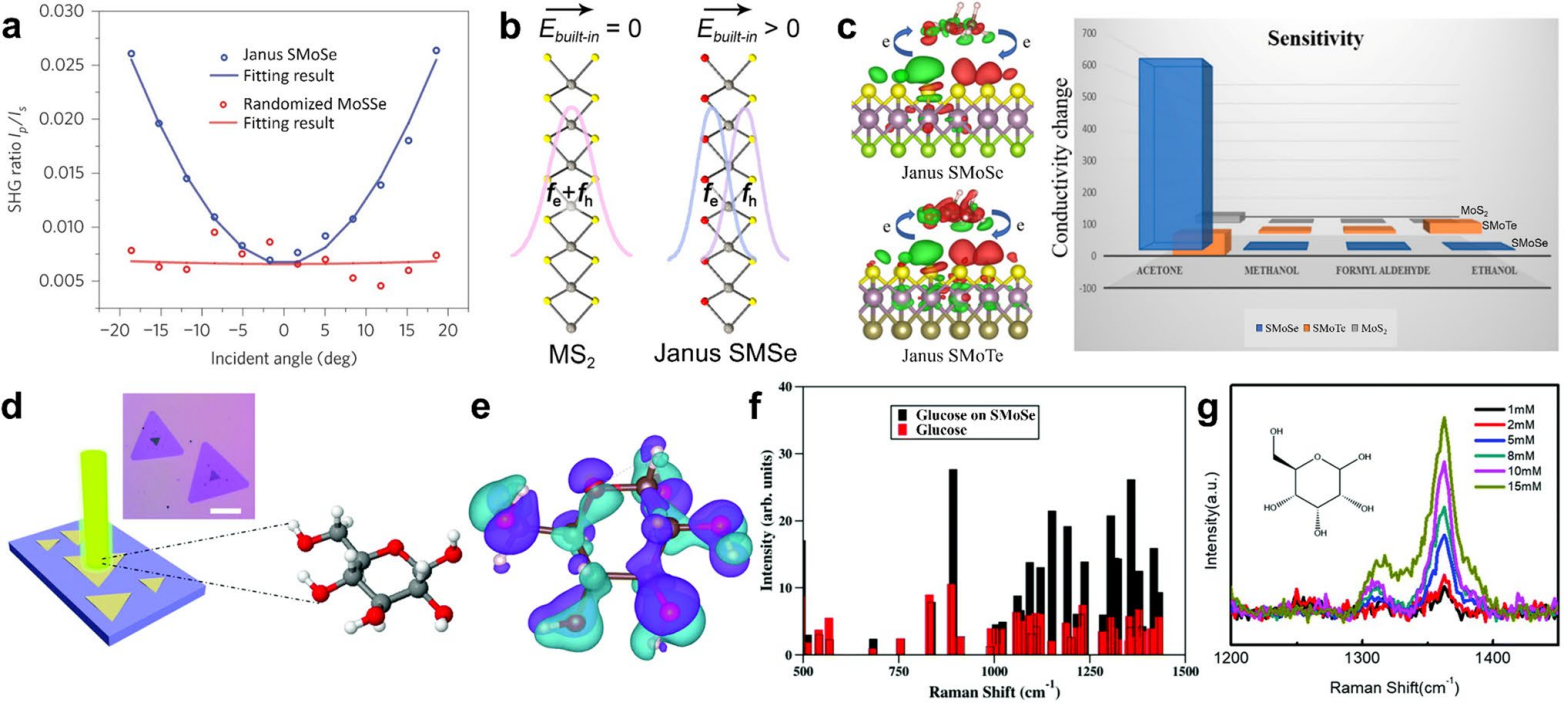
Characterization of built-in dipole moment in JTMD layers and ultrasensitive detection of organic molecules/biomolecules. **a** Angle-dependent SHG intensity ratio between p and s polarization in the Janus SMoSe and randomized alloy samples. The *I*_p_/*I*_s_ ratio (blue circles) increases symmetrically with more tilted incidence and is fitted well by an angle-dependent SHG model (blue curve). The *I*_p_/*I*_s_ ratio (red circles) undergoes almost no change as the incident angle varies, and the flat fitting (red curve) suggests a negligible out-of-plane dipole. Reprinted with permission from Ref. [50]. Copyright 2017, Nature Publishing Group. **b** Schematics of the crystalline structure (side view) of MS_2_ and Janus SMSe (*M* = Mo, W) and the corresponding wave functions of electron (f_e_) and hole (f_h_) in excitons in each material. Reprinted with permission from Ref. [174]. Copyright 2021, American Chemical Society. **c** Calculated electron density difference (EDD) plots of acetone adsorption on S layer of Janus SMoSe and SMoTe monolayers. Green and red represent the regions of electron depletion and accumulation, respectively. Reprinted with permission under a Creative Commons Non-Commercial No Derivative Works (CC-BY-NC-ND) Attribution License from Ref. [184]. Copyright 2020, American Chemical Society. **d–g** SERS-based ultrasensitive detection of glucose by Janus SMoSe: **d** optical image of Janus SMoSe and schematic of laser irradiation of glucose on Janus SMoSe for SERS characterization. The scale bar is 20 μm. **e–f** DFT calculations for the glucose molecule on the Janus substrate. **e** Charge distribution in glucose. The light blue and the purple regions show the electron cloud distribution in the isolated single glucose molecule and in anchored glucose on Janus SMoSe, respectively. Their distinguishing shapes indicate the drastic charge redistribution after glucose is adsorbed on monolayer Janus SMoSe. **f** Calculated Raman peaks for isolated glucose and anchored glucose on Janus SMoSe. **g** Concentration-dependent Raman spectra of glucose on Janus SMoSe. The integrated peak intensity increased linearly with elevated glucose concentration in solution. Reprinted with permission from Ref. [185]. Copyright 2020, Royal Society of Chemistry

***Unique Properties and Promising Application of JTMDs:*** The in-plane symmetry breaking of Janus XMY and the different electronegativities of the X and Y atoms result in a built-in vertical dipole moment in 2D JTMDs, providing an important platform for fabricating next-generation high-performance devices with applications in exciton dynamics, dipole interaction, spintronics, and piezoelectricity [50, 174]. Figure 17a shows that angle-dependent optical second harmonic generation (SHG) characterization of Janus SMoSe and random MoSSe alloy confirms the breaking of mirror symmetry and the existence of vertical dipoles in Janus SMoSe monolayers [50]. Interestingly, the wave functions of electrons and holes are separated under the built-in dipole moment in 2D JTMDs (Fig. 17b), which prolongs the exciton radiative recombination lifetime compared with binary TMDs and improves the efficiency and performance of JTMDs-based photovoltaic and photoelectric devices [174].

The presence of built-in dipole moments in JTMDs enhances electronic interactions between JTMDs and organic molecules. In Yeh’s study, the gas sensitivity of 2D TMDs and JTMDs to various volatile organic compounds (such as acetone, methanol, and formyl aldehyde) was investigated through DFT calculations [184]. The results showed that the adsorption of acetone molecules significantly altered the bandgaps of JTMDs compared to binary TMDs, resulting in a conspicuous change in conductivity for the ultrasensitive detection of acetone gas (Fig. 17c). This modulation/optimization of the electron transfer process can be attributed to the dipole interaction between the internal dipole moment of JTMDs and the polarized gas molecules. In another study, CVD-fabricated Janus SMoSe monolayers were utilized to detect various biomolecules (glucose and dopamine) via surface-enhanced Raman scattering (SERS) (Fig. 17d) [185]. The dipole moments provided by Janus SMoSe polarize dipoles in glucose and redistribute abundant charge to significantly enhance the Raman vibrational intensity of glucose (Fig. 17e, f). As a result, Janus SMoSe demonstrated a Raman enhancement factor of up to 10^5^ for glucose with a good linear detection relationship within a concentration of 1–10 mM (Fig. 17g).

In addition to the inherent vertical dipole moments present in JTMDs, the formation of the Janus structure in 2D TMDs changes their crystal and band structures. First-principles calculations suggest that the Janus SMoSe monolayer has an electron carrier mobility of 73.8 cm^2^ V^−1^ s^−1^ and a hole carrier mobility of 157.2 cm^2^ V^−1^ s^−1^ [186]. However, layer-stacked Janus SMoSe exhibits an electron carrier mobility of 1,194 cm^2^ V^−1^ s^−1^ in bilayer SMoSe structures and an extremely high hole carrier mobility of 5,894 cm^2^ V^−1^ s^−1^ in trilayer SMoSe structures. This layer-dependent enhanced carrier mobility can be explained by changes in deformation potential and elastic modulus. Lu’s work computationally and experimentally demonstrated that Janus SMoSe has much lower overpotential and hydrogen adsorption-free energy in HER compared to MoS_2_ and MoSe_2_ [51]. Additionally, Idrees et al. investigated the optical and electronic properties of JTMD monolayers and their heterostructures through hybrid DFT calculations [187]. The results indicated that these monolayers and heterostructures have strong optical absorption in the visible to ultraviolet regions, with SeWTe, SMoTe, and SWTe monolayers achieving an absorption efficiency of ~ 80% in the visible, infrared, and ultraviolet regions. It was also revealed that the water reduction and oxidation potentials for all JTMD monolayers at pH = 0 are located between their valence and conduction band edges, making them promising candidates for water splitting.

## Summary and Outlooks

The extensive research and exploitation of binary TMDs have made it increasingly important to tune and improve their properties for potential applications in modern realms. Atomically substitutional engineering is an exclusive strategy for TMDs that combines diverse TMDs with different structural and electronic properties to form ternary or quaternary uniform solid solutions with special crystal phases, continuously tunable bandgaps, tailored band alignment/structure, and exotic physical phenomena (such as superconductivity and Weyl semi-metallicity). The good miscibility of TMDs allows for arbitrary mixing percentages in ATMDs and forms an internal contact in monolayers, making atomic substitution clearly superior to elemental doping and hybridization with other nanomaterials. Compared to binary TMDs, ATMDs can achieve enhanced/improved performances in a wide range of advanced applications such as electronic devices, photoelectronic sensing, electrocatalysis, and photoelectronic conversion. The emergence of 2D ATMDs has introduced a range of new members to the family of 2D nanomaterials, offering a highly competitive and flexible platform for numerous contemporary industrial applications.

In recent years, rapid progress has been witnessed in the facile synthesis, outstanding properties, and novel applications of ATMD layers. However, there are still some challenges in their exploitation and investigation. For instance, most current studies focus on the fabrication of ternary TMDs, with only a few quaternary TMDs reported [33, 75, 188]. Generally, 2D ATMDs containing more elements should exhibit a wider range of property modulation, more functions, and even unprecedented properties compared to ternary TMDs [189]. To advance in this direction, more research efforts are directed toward the synthesis of high-quality quaternary TMDs. They refer to not only MNXY-type TMDs mentioned earlier but also quaternary TMDs with additional cationic or anionic substitutions. Examples of these emerging materials include MXYZ-type MoS_2(1−*x*−*y*)_Se_2*x*_Te_2*y*_ and MNOX-type Mo_1−*x*−*y*_W_*x*_V_*y*_Se_2_ [104, 190]. These TMDs offer more flexibility in tuning their properties and demonstrate superior performance for various applications. Furthermore, recently discovered multinary TMDs with non/sub-stoichiometric phases, obtained through tailored experimental conditions, demonstrate unique physical properties and impressive performance [191, 192]. For instance, Li_x_MoS_2_ exhibits superior thermal conductivity, while Fe_x_NbS_2_ displays an induced antiferromagnetic order [192]. These materials hold great promise and should be extensively studied and appreciated in future research. In terms of synthetic methods, CVD and hydro/solvothermal methods have proven to be the most effective for fabricating high-quality 2D ATMDs, but the quality of products is critically dependent on the types/states of the precursors, experimental conditions, and even the reaction apparatus. In the future, more attempts should be made to simplify the synthesis of 2D ATMDs and obtain high-quality products under mild conditions. One can realize the production of highly uniform ATMDs in CVD by spin-coating liquid precursors onto substrates [193, 194]. The large-scale manufacturing of ATMDs on wafers can be accomplished by designing wafer modules within CVD furnaces and establishing pathways for precursor supply [103, 104]. The crystalline quality of these materials can be improved by carefully selecting specific substrates. For example, it has been demonstrated that Re-based TMDs exhibit an improved lattice matching with mica compared to SiO_2_/Si substrates, leading to a better crystalline quality [195, 196]. Regarding the synthesis of Janus ATMDs, only Janus SMoSe and SWSe have been successfully fabricated, although various 2D JTMDs have been theoretically simulated to exhibit good thermodynamic stability. Further research is needed to develop effective strategies to synthesize a broader range of Janus ATMDs with tailored properties for various applications.

The utilization of 2D ATMDs is still in the initial stages, leaving ample room to explore their enhanced/novel applications. Atomic substitution of binary TMDs has proven effective in modulating the internal lattices to achieve tunable Raman scattering and inducing lattice disorder and phase transitions through the creation of atomic vacancies. Therefore, atomically substitutional engineering is a promising strategy to enhance the SERS performance of 2D ATMDs [197]. Additionally, multinary TMDs can alter the band alignment/structure, defect state energy level, carrier density/type, and broaden spectral response, making ATMD layers desirable channel materials for developing advanced FETs and photodetectors. Moving forward, it is crucial to direct the rapid development of 2D ATMDs for use in logic devices, photovoltaic cells, light-emitting diodes, and other related areas [42, 198, 199]. Specifically, there should be a widespread promotion of ATMDs with tailored spectral response ranges and band alignments in the fields of photodetection, photocatalysis, and self-powered optoelectronic devices [42, 164, 200]. Additionally, emphasis should be placed on ATMDs with crystalline phase transition capabilities for highly sensitive sensing and detection of multifarious trace molecules [3, 197]. Furthermore, recent studies have demonstrated that specific atomic substitution in binary TMDs, such as V_*x*_Mo_1−*x*_Se_2_ and Re_*x*_Mo_1−*x*_Se_2_, can induce magnetism, thereby broadening the avenues for fundamental physics research and the exploitation of intriguing spintronic devices [47, 86]. This requires further optimization of device structure, fabrication of more 2D ATMDs with new/multi-components, and even hybridization/coupling of them with various nanostructures beyond 2D ATMDs. Ongoing research in these areas will lead to the practical realization of advanced applications of ATMDs.
